# CFPR-YOLO: chili flower pose estimation for robotic pollination in unstructured environments

**DOI:** 10.3389/fpls.2026.1861155

**Published:** 2026-06-05

**Authors:** Minqiu Kuang, Yushi Wang, Xiaojian Li, Dawei Liu, Yang Xiang, Feng Liu, Xiangjun Zou, Fangping Xie, Yuxuan Zhang, Xu Li

**Affiliations:** 1 College of Electrical and Mechanical Engineering, Hunan Agricultural University, Changsha, China; 2 Foshan-Zhongke Innovation Research Institute of Intelligent Agriculture and Robotics, Foshan, China; 3 Hunan Provincial Key Laboratory of Intelligent Agricultural Machinery Equipment, Changsha, China; 4 College of Horticulture, Hunan Agricultural University, Changsha, China; 5 College of Intelligent Science and Engineering, Beijing University of Agriculture, Beijing, China; 6 Department of Computer and Electrical Engineering, Mid Sweden University, Sundsvall, Sweden

**Keywords:** chili flower, deep learning, edge computing, intelligent horticulture, plant phenotyping, pose estimation, precision pollination

## Abstract

Agricultural engineering informatics is playing an increasingly important role in enabling intelligent perception, decision-making, and automated operations in modern horticultural production systems. Within this context, accurate visual perception of reproductive structures is essential for agricultural informatization tasks such as flowering-stage monitoring, precision pollination, and information-driven fruit-set management in chili cultivation. However, reliable detection and pose-aware recognition of chili flowers remain challenging because of small target size, dense distribution, foliage occlusion, and illumination variability in natural or semi-controlled environments. To address these challenges, this study proposes a lightweight and robust edge vision framework, termed CFPR-YOLO, for chili flower detection and pose-aware perception under complex agricultural conditions. Built upon an improved YOLOv11n architecture, the proposed framework incorporates EfficientFormerV2 to strengthen global-context feature extraction, a C3k2_EMA module to enhance localization of small and occluded targets, and Poly-Scale Convolution (PSConv) to preserve structural details while reducing computational redundancy. In addition, a lightweight attention mechanism is introduced to improve feature discrimination in cluttered backgrounds. Experimental results on both self-constructed and generalization datasets show that the proposed method achieves a precision of 92.6%, a recall of 86.8%, and an mAP50 of 92.1% with only 7.26 M parameters. The framework also demonstrates strong robustness and generalization across different chili varieties. When deployed on an edge computing platform (NVIDIA Jetson AGX Orin), the model achieves real-time inference at 39.5 FPS. Furthermore, validation experiments under controlled indoor conditions show that the proposed framework can effectively support simulated pollination tasks, achieving a success rate of 90.0% for upwardfacing flowers. These results indicate that CFPR-YOLO provides an effective visual perception solution for agricultural engineering informatics-oriented pollination systems and offers practical potential for precision pollination and intelligent fruit-set management in horticultural production.

## 1 Introduction

### 1.1 Research background

Chili is an economically important horticultural crop, and its breeding and production are closely associated with yield improvement, quality enhancement, and market competitiveness Lin et al. (2023); Zou et al. (2025). In modern protected and semi-controlled cultivation systems, not only total yield but also the uniformity of fruit set has become increasingly important, because flowering-stage consistency directly affects downstream fruit development, grading stability, and market value. From the perspective of agricultural engineering informatics, this challenge is no longer merely a biological or agronomic issue; rather, it requires effective acquisition, interpretation, and utilization of field information to support timely sensing, precise intervention, and intelligent operation throughout the flowering stage.

Agriculture is currently undergoing a profound transition driven by labor shortages, climate variability, and resource constraints. Under such conditions, intelligent cultivation and management strategies integrating sensing, data processing, automated control, and robotic execution are becoming essential for sustainable productivity improvement Junyang et al. (2026); Li et al. (2025c); Tang et al. (2023); Fatchurrahman et al. (2025); Lu et al. (2026b). As digital agriculture continues to advance, agricultural engineering informatics is increasingly serving as the core enabling paradigm for converting raw field observations into actionable intelligence through perception models, edge computing, and decision-oriented automation Zhang et al. (2025); Bhattarai et al. (2025); Zhang et al. (2026c, Zhang et al., 2026b). Within this context, flowering-stage monitoring has become a critical information acquisition task, because accurate perception of reproductive organs is fundamental to precision operations such as robotic pollination, reproductive-status assessment, and fruit-set management. Among these applications, automated pollination has attracted increasing attention as a promising technological route for improving fruit set consistency and operational efficiency in controlled-environment horticulture Yang et al. (2023); Li et al. (2023).

Autonomous pollination technologies are important not only because they can alleviate labor dependence and compensate for the decline of natural pollination services, but also because they can provide more standardized, repeatable, and information-driven interventions during the reproductive stage of crop growth Ahmad et al. (2024). For chili production, such precision is especially relevant, since flower orientation and accessibility directly influence whether pollination can be executed effectively and consistently. In this sense, flower perception is not simply an isolated vision task; it is a key front-end component in an agricultural engineering informatics pipeline that links visual sensing, information extraction, robotic decision-making, and operation planning. However, reliable deployment of chili pollination systems in natural or semi-controlled environments remains challenging. Illumination fluctuations, leaf and branch occlusions, dense target distribution, and subtle inter-class pose differences significantly complicate accurate flower recognition and orientation discrimination. Therefore, robust pose-aware visual perception is a necessary prerequisite for enabling precise, repeatable, and information-driven pollination operations Deng et al. (2020); He et al. (2025); Redmon et al. (2016).

### 1.2 Related works

Recent advances in computer vision have created favorable conditions for building such perception systems Lu et al. (2026a); Han et al. (2026); Li et al. (2025a); Zhang et al. (2026d); Lu et al. (2026c); Zhang et al. (2026a). In particular, object detection frameworks represented by the YOLO family, originally developed on convolutional neural networks (CNN) Han et al. (2022) and more recently influenced by Transformer-based architectures Li et al. (2024), have shown strong potential for real-time agricultural vision applications. Due to their favorable balance between accuracy and computational efficiency, these methods have been increasingly applied in agricultural scenarios involving crop monitoring, organ recognition, and robot-oriented perception Li et al. (2021); Shang et al. (2024); Wei et al. (2024); Zhang et al. (2024). These developments have laid an important technical foundation for visual information extraction in complex agricultural environments, particularly when real-time response, edge deployment, and perception-driven operations are required.

In the specific domain of crop flower recognition, a series of encouraging studies have been reported. Lyu et al. (2023) optimized YOLOv7 and achieved a recognition accuracy of 99.4% for pear blossoms. Li et al. (2022) proposed a cascaded deep learning framework that reduced occlusion interference between tomato flowers and fruits, reaching an accuracy of 87.92% for small-flower recognition. Shang et al. (2022) combined YOLOv5l with Euclidean distance metrics and obtained an mAP of 91.60% for multi-class apricot blossom detection. Wang and Zhang (2025) developed an improved YOLOv5s model for apple blossom detection and reported an AP of 96.40% for white blossoms, demonstrating the feasibility of detecting visually similar floral targets. Liu and Li (2025) proposed CRV-YOLO and achieved an AP of 96.9% in apple blossom detection. For densely distributed small targets, Liu et al. Kuang et al. (2025a) improved YOLOv8n and achieved an mAP50 of 92.4% for apricot blossoms. In addition, Kuang et al. (2026b); Yu et al. (2022); Kuang et al. (2026c) developed lightweight YOLO-based detection models with strong performance in complex environments, offering useful architectural insights for edge-oriented agricultural perception. Recent studies have further promoted the application of advanced YOLO variants in agricultural perception tasks. Zhao et al. Zhao et al. (2025a) proposed UDD-YOLO for crop detection under complex field environments, improving robustness against illumination variation and dense occlusion. You et al. You et al. (2025) introduced Rose-YOLO for rose organ detection, achieving favorable accuracy-speed trade-offs through lightweight feature aggregation. To further enhance long-range dependency modeling, Zhao et al. Zhao et al. (2025b) developed Rose-Mamba-YOLO by integrating the Mamba mechanism into the YOLO framework, significantly improving feature representation capability in cluttered agricultural scenes. Li et al. Li et al. (2025b) proposed Succulent-YOLO for dense succulent plant detection and demonstrated improved small-target localization performance. Liu et al. Liu et al. (2026) presented FP-YOLO for agricultural object perception in challenging environments, achieving enhanced detection accuracy while maintaining computational efficiency. In addition, Tao et al. Tao et al. (2025) introduced a diffusion-assisted YOLO framework that improved feature discrimination and robustness under occlusion and complex backgrounds. These studies demonstrate that recent YOLO variants are increasingly emphasizing the balance among lightweight deployment, feature extraction capability, and robustness in complex agricultural environments. However, most existing methods still primarily focus on generic object detection tasks, whereas the present study further addresses flower orientation recognition for pollination-oriented robotic perception. Moreover, our method emphasizes not only detection accuracy but also the practical integration of perception outputs into downstream precision pollination operations. Kuang et al. (2025b, Kuang et al., 2026a); Lee et al. (2025) further combined object detection and classification to support flower detection, flowering-stage identification, and posture recognition for tomato blossoms, thereby illustrating the practical value of integrating visual perception with automated agricultural operations.

Despite this progress, several limitations remain before such methods can fully satisfy the requirements of agricultural engineering informatics-oriented pollination systems. First, most existing studies still focus primarily on two-dimensional target detection, whereas flower orientation recognition is more directly relevant to robotic manipulation and precision pollination. Second, chili flowers pose a particularly difficult perception problem because of their small size, dense distribution, low contrast against foliage, and frequent occlusion, which jointly reduce the reliability of orientation discrimination. Third, the trade-off between recognition accuracy and computational efficiency remains unresolved in many studies. Models with strong feature extraction ability often introduce higher parameter counts and computational burdens, making them less suitable for resource-constrained edge devices. Fourth, many current studies stop at image-level detection evaluation and do not sufficiently validate whether the extracted visual information can effectively support downstream operations. As a result, the connection between perception performance and agricultural task effectiveness remains weak. In other words, the central issue is not only whether flowers can be detected accurately, but whether perception outputs can be transformed into operationally meaningful information for intelligent agricultural systems. This gap is especially critical in pollination tasks, where perception must ultimately serve decision-making and action execution.

### 1.3 Our contributions

To address these challenges, this study proposes a lightweight deep learning framework, termed CFPRYOLO, for chili flower detection and pose-aware perception in complex agricultural environments. The proposed method is designed from an agricultural engineering informatics perspective, in which visual sensing is treated as the front end of an integrated perception-to-action pipeline for intelligent pollination. Specifically, the framework aims to enhance the accuracy and robustness of flower recognition under small-target, occluded, and illumination-variable conditions while maintaining the computational efficiency necessary for edge deployment and real-time operation. Beyond image-level evaluation, the proposed method is further validated in simulated pollination tasks under controlled indoor conditions so that its practical value can be assessed in relation to downstream operational requirements. By linking pose-aware visual perception with pollination-oriented execution, this study seeks to provide a more informative and deployable technical route for intelligent flowering-stage monitoring and precision pollination management in chili production systems. This work contributes to the development of visual perception systems for robotic pollination in agricultural environments. Rather than focusing on fundamentally new model architectures, this study emphasizes system-level integration and task-oriented design. The main contributions are summarized as follows:

- An overview of a lightweight perception-to-action framework for chili flower detection and pose-aware perception, designed for edge deployment under controlled agricultural conditions;
- A task-driven integration and adaptation of multiple efficient components, including EfficientFormerV2, C3k2_EMA, PSConv, and SimAM, to improve robustness in small-target detection, occlusion handling, and pose discrimination under the tested conditions;
- A validation strategy that combines image-level evaluation with real-world robotic platform validation under controlled experimental conditions, enabling assessment of the proposed method in relation to downstream execution tasks;

The remainder of this paper is organized as follows. Section 2 describes the materials and methods, including dataset construction, data augmentation, and the architecture of the proposed CFPR-YOLO framework. Section 3 presents the experimental results and discussion, including ablation studies, comparative analyses, visualization results, and validation under simulated pollination conditions. Section 4 concludes the paper and discusses future research directions.

## 2 Materials and methods

### 2.1 Image collection and dataset preprocessing

The chili flower dataset used in this study was collected from the vegetable cultivation base of Hunan Agricultural University, situated in Furong District, Changsha City, Hunan Province. Image acquisition was conducted using a Canon R50 color intelligent digital camera between May and June 2024. To ensure high-quality data, images were captured during the intervals of 6:00-11:00 a.m. and 5:00-7:00 p.m., when illumination conditions were most favorable for obtaining clear and feature-rich images, which also correspond to the optimal pollination periods under agronomic practices. The experimental material consisted of the 8214upup mutant, a chili variety cultivated in the laboratory that typically exhibits an upward growth orientation. All collected images were of high resolution.

**Figure 1:**
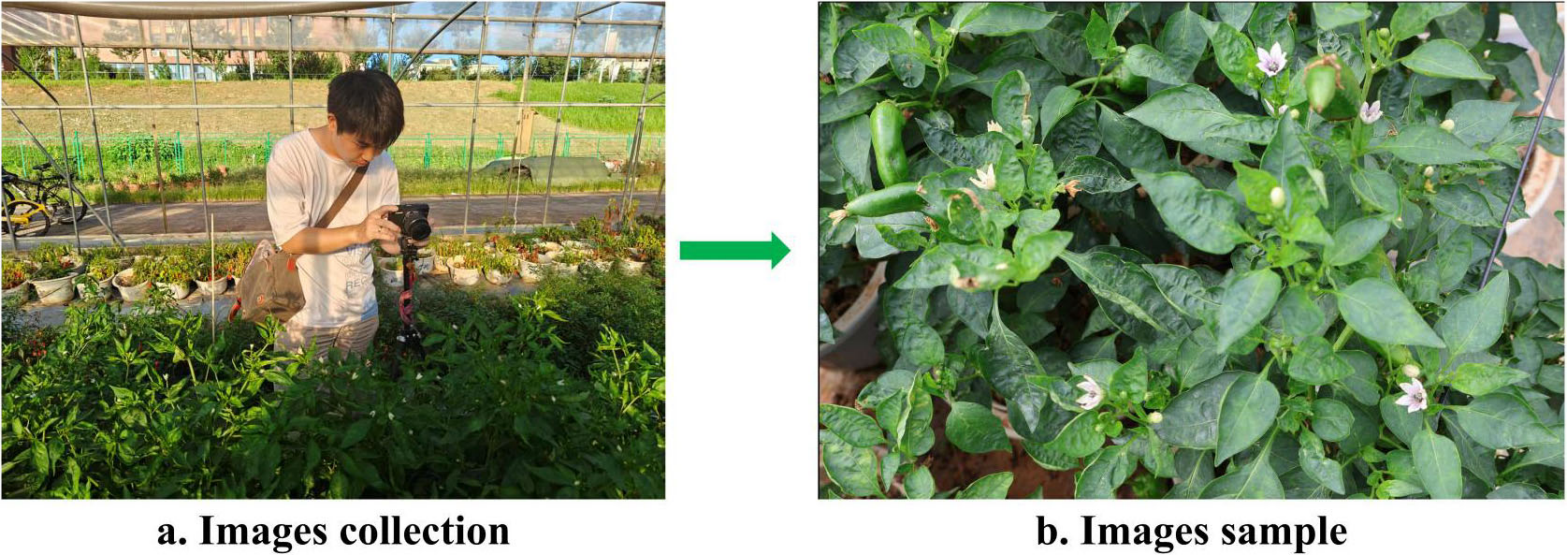
Chili flower image collection process. **(a)** Field images collection scene: an author collecting images of chili flowers in a greenhouse using a digital camera. **(b)** Representative image sample: a close-up view of chili flowers, buds, and developing fruits in a natural growth environment. The person in the image is one of the authors of this paper, and there is no copyright dispute regarding the use of this image.

During image collection, professional digital cameras operating in burst mode were positioned overhead and used to capture chili flowers from multiple angles, focal lengths, and time points. However, under natural environmental influences such as wind and uneven light distribution, even though the dominant growth orientation of the 8214upup mutant is upward, four additional derived orientations, leftward, rightward, forward, and backward, were observed. As illustrated in **Figure 1**, these variations reflect the data acquisition process for the natural-environment chili flower dataset.

**Figure 2:**
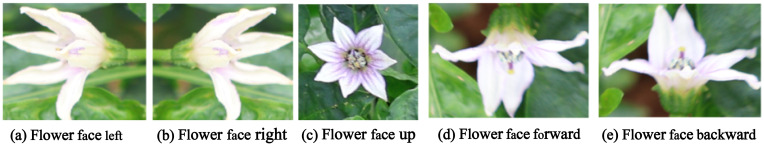
Five typical growth postures of field-collected chili flowers: **(a)** Flower facing left, **(b)** Flower facing right, **(c)** Flower facing upward, **(d)** Flower facing forward, **(e)** Flower facing backward.

To improve the generalization capacity of the dataset, image collection was carried out under diverse weather conditions, including transitions from cloudy to sunny and from overcast to light rainfall. Photographs were taken at varying distances, angles, and lighting conditions, resulting in a total of 6,073 images. After manual screening to remove blurred and severely occluded samples, a final raw dataset of 4,219 images was compiled. As shown in **Figure 2**, five representative postures of chili flowers captured under natural conditions are illustrated, namely left, right, upward, forward, and backward orientations. Specifically, **Figure 2a** depicts the leftward perspective, **Figure 2b** shows the rightward perspective, **Figure 2c** represents the upward front perspective, **Figure 2d** illustrates the forward perspective, and **Figure 2e** presents the backward perspective.

**Figure 3:**
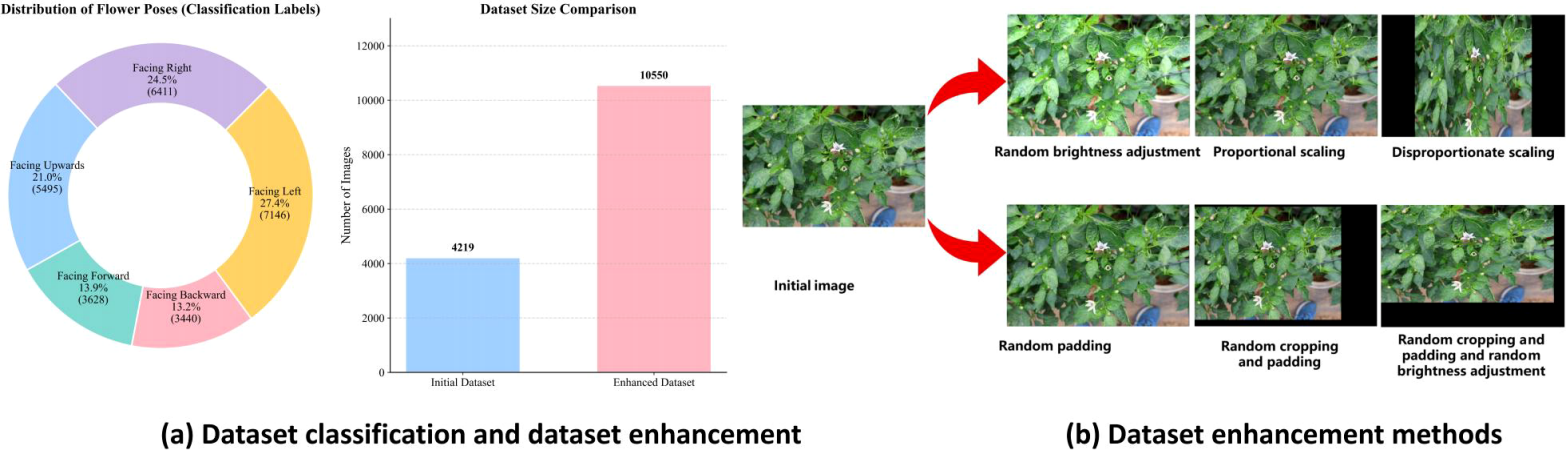
**(a)** Dataset classification and quantity statistics; **(b)** Data enhancement methods and example effects.

To strengthen the model’s generalization ability, this study applied data augmentation techniques, including random brightness adjustment, random scaling, random translation, and composite transformations, thereby expanding the dataset to 10,550 images. The collected data were then thoroughly annotated using the X-Anylabeling professional annotation software, which identified the positional regions of each flower along with their corresponding orientation categories (leftward, rightward, upward, forward, and backward). **Figure 3a** summarizes the labeling results, while **Figure 3b** illustrates examples of augmented chili flower, highlighting variations in color contrast, spatial positioning, and size ratio. Finally, the dataset was divided into a training set (8,440 images), a validation set (1,055 images), and a test set (1,055 images) in an 8:1:1 ratio for model development.

### 2.2 Technical route

**Figure 4:**
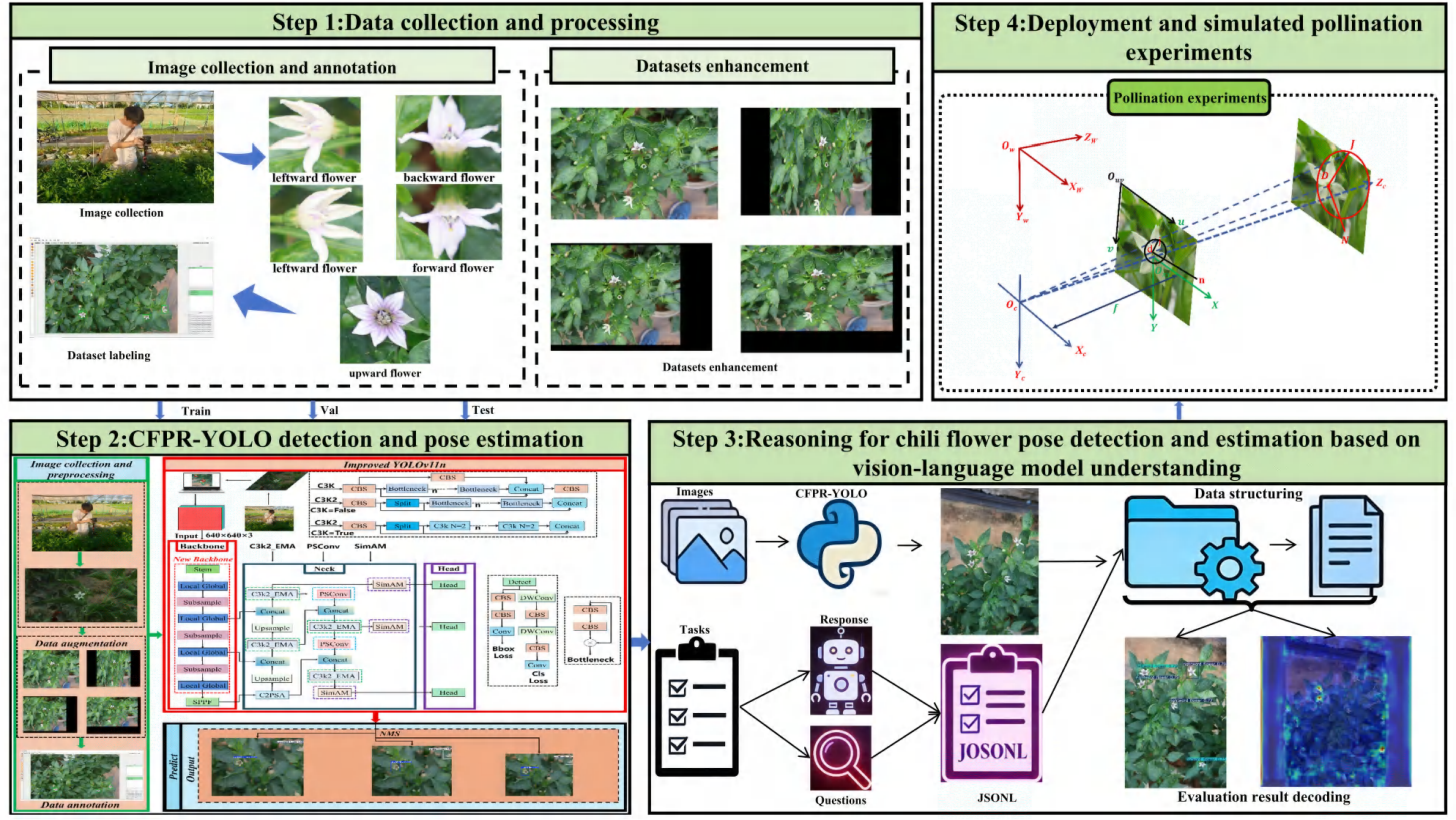
Overall research framework of the study.

The overall workflow of the proposed system is illustrated in **Figure 4**, which integrates data acquisition, visual perception, decision-making, and simulated operation into a unified framework. In the first stage, a high-diversity image dataset of chili flowers was constructed under varying illumination conditions, occlusion levels, and growth states. To improve model robustness, data augmentation strategies were applied to enhance the representation of small targets and complex backgrounds, thereby providing a reliable basis for feature learning in real-world environments. In the second stage, a lightweight detection and pose estimation model, CFPR-YOLO, was developed to accurately identify chili flowers and classify their orientations. By incorporating global context modeling and multi-scale feature enhancement mechanisms, the model improves sensitivity to subtle morphological characteristics while maintaining computational efficiency suitable for edge deployment. In the third stage, the detected flower regions and pose information were further processed to generate structured outputs that can be directly utilized for downstream tasks. This step enables the transformation of visual perception results into actionable spatial information, supporting precise positioning requirements for automated operations. In the final stage, the proposed framework was deployed on an embedded edge platform and evaluated through simulated pollination experiments in a controlled indoor environment. The perception results were mapped to spatial coordinates to guide robotic manipulation, allowing assessment of system performance under realistic operating conditions.

Overall, this workflow establishes a complete perception-to-action pipeline, enabling accurate flower recognition and pose estimation to support controlled pollination processes. From an agricultural perspective, improving the consistency and reliability of pollination operations may contribute to enhanced fruit set uniformity, which is closely related to the stability of fruit development and postharvest quality.

### 2.3 Chili flower pose estimation recognition model

YOLOv11, a new-generation real-time object detection algorithm, builds upon the architectures of YOLOv5 and YOLOv8. Compared with YOLOv8, YOLOv11 introduces several key enhancements. First, it improves the feature extraction module by replacing the C2f module with the C3k2 module, which increases the number of convolutional layers and incorporates an attention mechanism, thereby strengthening the ability to extract target features in complex environments and improving adaptability across diverse scenarios. Second, it enhances the attention mechanism by adding a class attention mechanism (C2PSA) layer after the SPPF module, allowing the model to adaptively focus on key feature regions and significantly improve feature representation accuracy. Third, it simplifies the detection head by substituting the conventional category detection convolution with a depthwise separable convolution (DWConv), reducing computational cost while ensuring efficient cross-channel information flow. Fourth, it optimizes the overall structural balance of the model by adjusting its depth and width parameters, achieving a dynamic trade-off between detection accuracy and processing speed.

**Figure 5:**
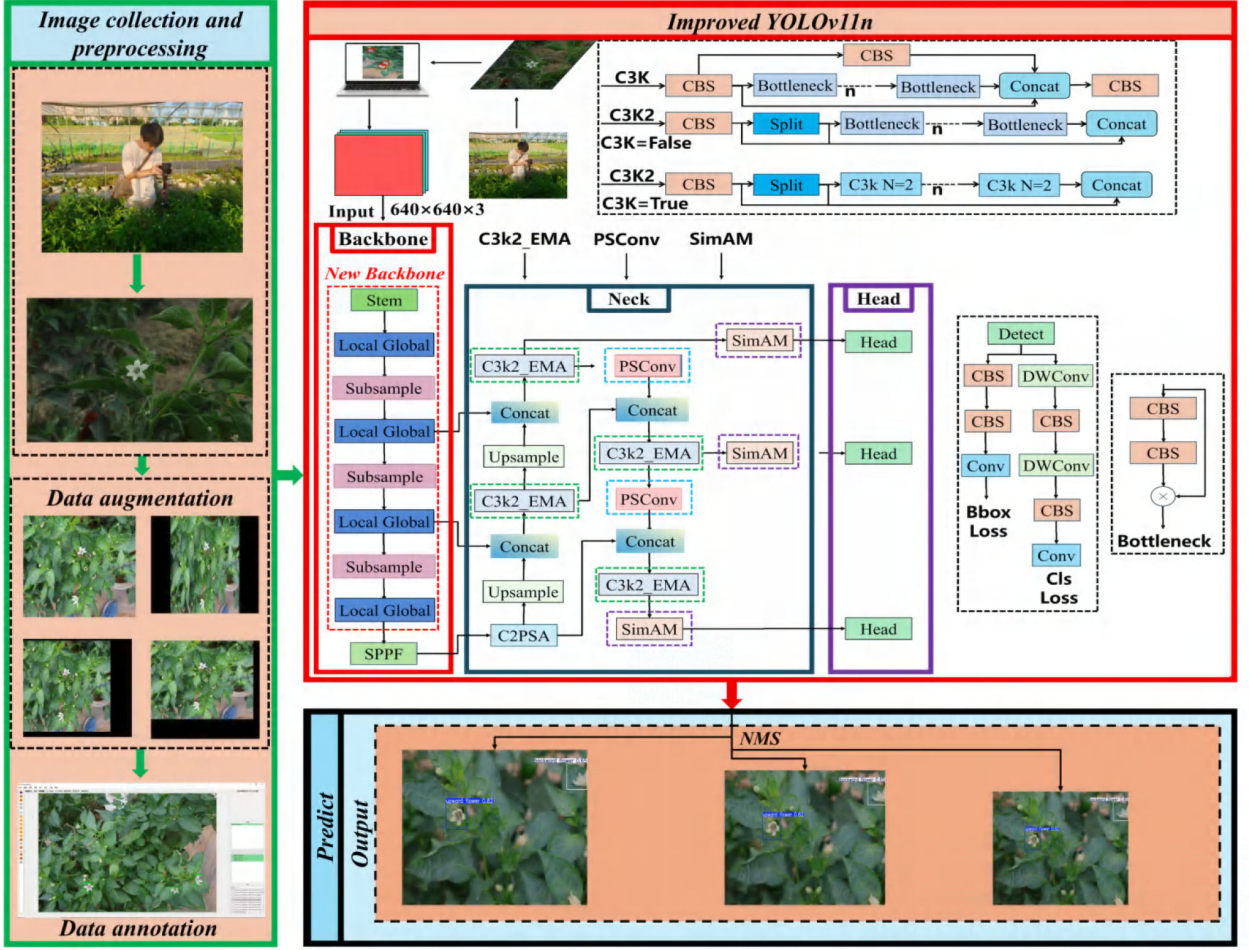
Network architecture of YOLOv11n and CFPR-YOLO model.

This study focuses on a small-target pose estimation and recognition algorithm designed for the intelligent pollination of chili plants in complex natural environments. Building on YOLOv11n, an improved chili flower pose estimation and recognition model, CFPR-YOLO, is proposed. The redesigned model reduces parameters and computational overhead while maintaining high detection accuracy through backbone replacement, enhancements to the C3k2 module, selective convolution substitutions, and the integration of a lightweight parameter-free attention mechanism. Specifically, CFPR-YOLO incorporates several key improvements: (1) EfficientFormerV2 is employed as the backbone network, effectively alleviating the challenges posed by complex lighting conditions. (2) The EMA attention mechanism is introduced into the neck network and combined with the C3k2 module to form the C3k2_EMA module, which adaptively improves pose recognition accuracy. (3) A novel PSConv downsampling module is designed to optimize feature sampling, reducing computational complexity while preserving detection accuracy. (4) The lightweight SimAM attention mechanism is integrated, enabling precise capture of essential features without adding extra parameters, thereby enhancing the detection of small targets. The structural differences between the original and improved models are shown in **Figure 5**, which highlights essential components such as the Conv standard convolution, DWConv, SPPF spatial pyramid pooling module, C3k2 module, upsampling layers, and Concat cross-layer feature fusion, as well as the newly introduced C3k2_EMA module and SimAM mechanism.

#### 2.3.1 EfficientFormerV2 Backbone Network

**Figure 6:**
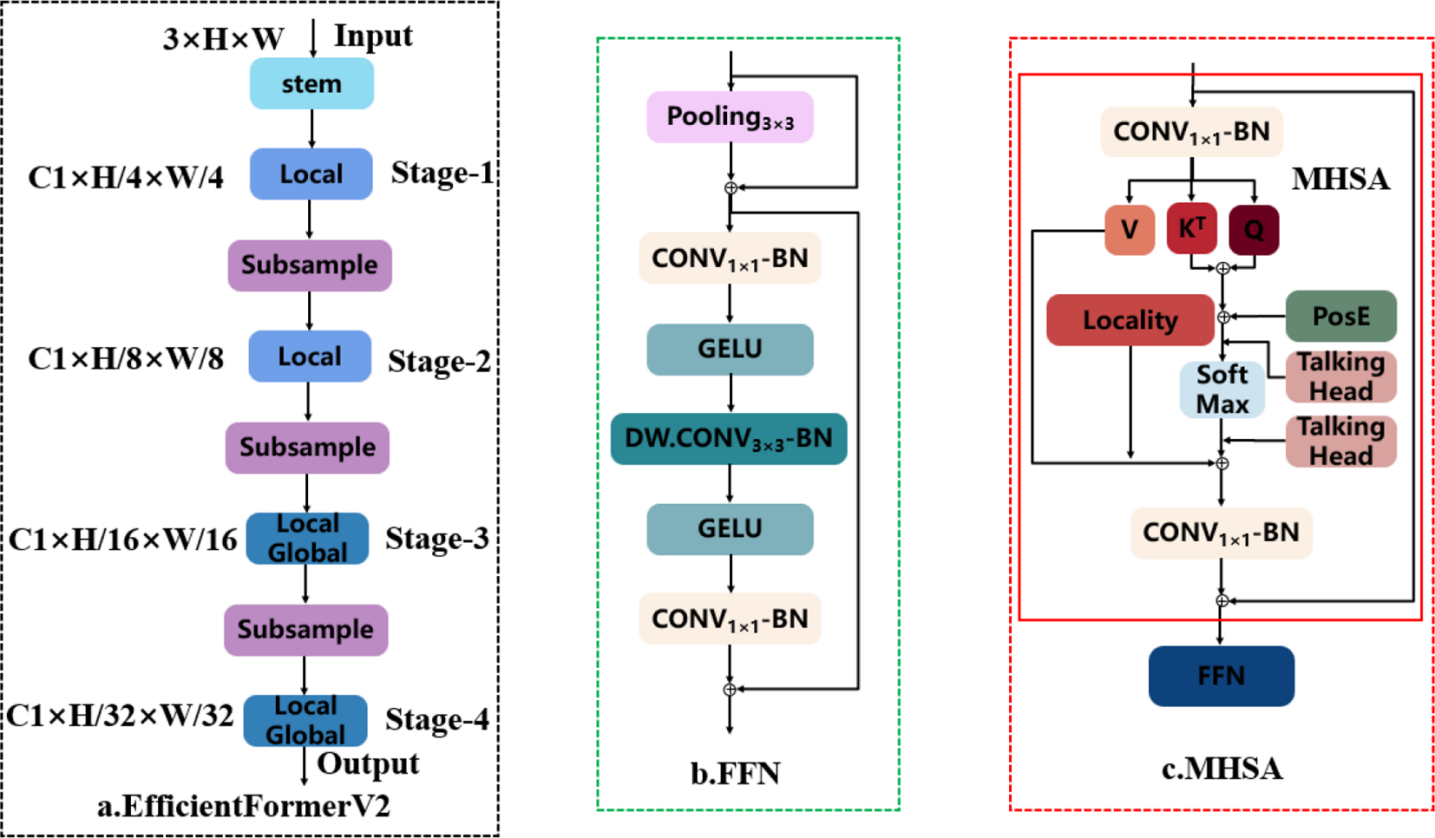
EfficientFormerV2 backbone network structure.

EfficientFormerV2, a representative model of the Transformer architecture, is designed to optimize inference latency while maintaining high accuracy, thereby significantly improving computational efficiency. For this reason, the present study adopts EfficientFormerV2 to replace the original New CSP-Darknet53 as the backbone network, with the goal of strengthening the model’s ability to extract chili flower posture features. The network structure of EfficientFormerV2, illustrated in **Figure 6**, follows a fourstage hierarchical design that sequentially generates feature maps at resolutions of 1*/*4, 1*/*8, 1*/*16, and 1*/*32 of the input image.

The input chili flower image is first pre-processed through the stem module, which consists of two 3 × 3 convolution kernels with a stride of 2. The detailed computation process is expressed in Equation 1:

(1) $$X_{i|i=\;1,j=1,\frac{H}{4},\;\frac{w}{4}}\;=\;stem\;\left(X_{0}^{B,3,H,W}\right)$$

In the formula, *X*_0_ represents the input image tensor, where *B* denotes the batch size, *C* the number of channels, and *H* and *W* the height and width of the feature map, respectively. The variable *i* indicates the network layer number, while *j* corresponds to the input variable. As the first component of the network, the stem module performs initial feature extraction and spatial dimension compression through two down-sampling operations, thereby establishing the foundation for subsequent multi-scale feature fusion.

In the first two stages, feed-forward neural networks (FFN) are uniformly configured to preserve the detailed information of the chili feature map at high resolution. This FFN design introduces an innovative approach by employing DWConv to replace the local token mixer traditionally used in explicit residual connections, while improving information transmission through cross-FFN residual connections, as expressed in Equation 2:

(2) $$X_{i+1,j}^{B,C_{j},\frac{H}{2^{j+1}},\frac{W}{2^{j+1}}}=S_{i,j}\cdot FFN^{C_{j}}\left(X_{i,j}\right)+X_{i,j}$$

In the final two stages, the local FFN is combined with global multi-head self-attention (MHSA) modules to enable collaborative extraction of both detailed and global features of chili flowers, as expressed in Equations 3, 4:

(3) $$X_{i+1,j}^{B,C_{j},\frac{H}{2^{j+1}},\frac{W}{2^{j+1}}}=S_{i,j}\cdot MHSA\left(Proj\left(X_{i,j}\right)\right)+X_{i,j}$$

(4) $$MHSA\left(Q,K,V\right)=Softmax\left(Q\cdot K^{T}+ab\right)\cdot V$$

In the formula, *S_i,j_*denotes a learnable scale factor, while *C_j_*(channel dimension) and *E_i,j_*(expansion ratio) jointly determine the structural attributes of the FFN. The MHSA generates queries (*Q*), keys (*K*), and *V* through a linear projection layer, optimizing the attention mechanism by incorporating a learnable positional encoding bias. Furthermore, its performance is enhanced without increasing model complexity through two strategies: (1) applying a 3 × 3 convolution to integrate local information into the value matrix, and (2) introducing a fully connected layer across head dimensions (“talking head”) to strengthen cross-head interactions. Together, these measures enable efficient fusion of local and global features of chili flowers.

#### 2.3.2 C3k2_EMA feature extraction module

**Figure 7:**
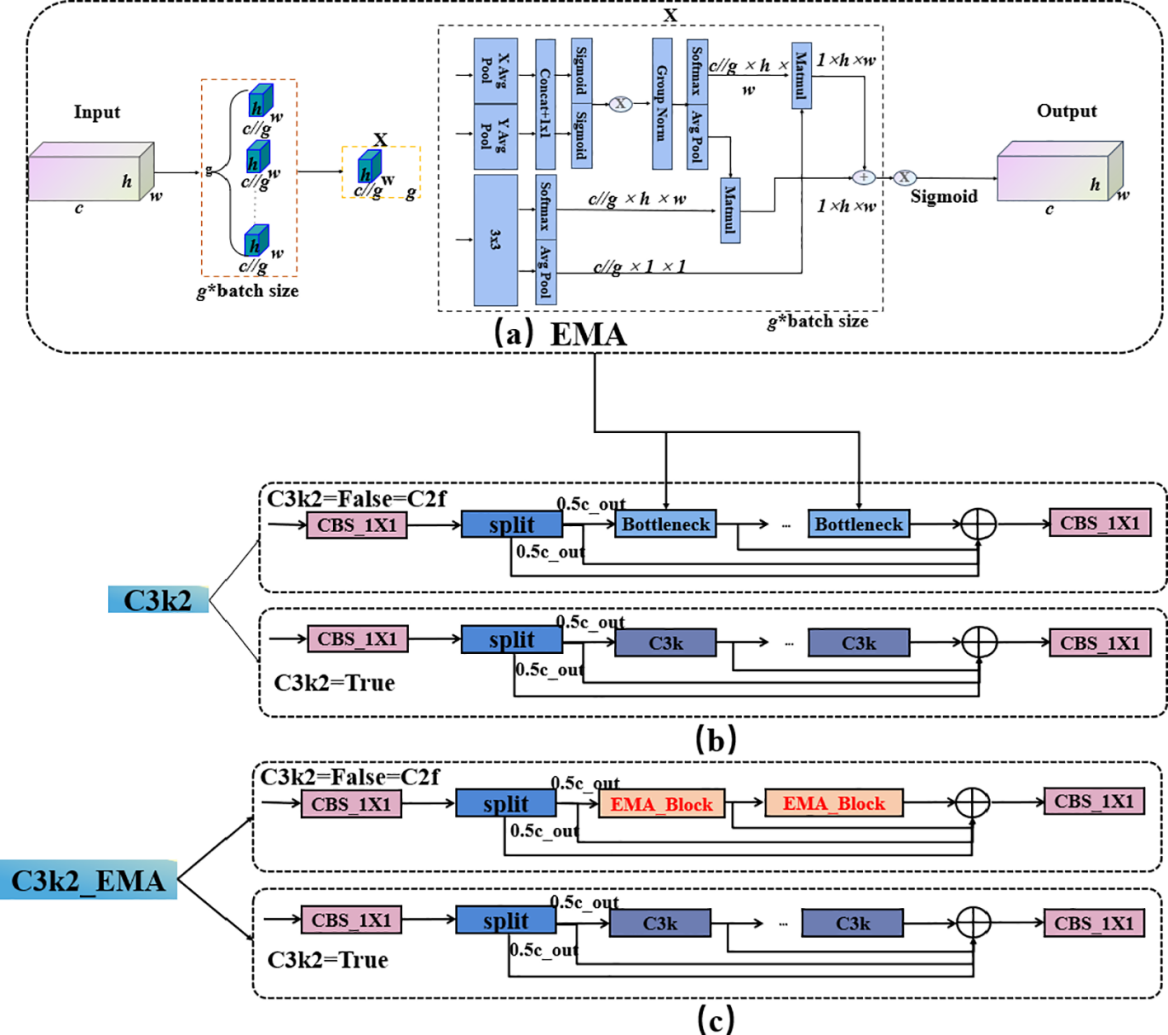
Structure diagram of the proposed C3k2_EMA module. **(a)** Detailed architecture of the Efficient Multi-scale Attention (EMA) module. **(b)** Schematic of the standard C3k2 module, showing its two variants: C2f (when C3k2=False) and C3k (when C3k2=True). **(c)** Schematic of the improved C3k2_EMA module, where the Bottleneck blocks in the original C2f variant are replaced with the proposed EMA blocks to enhance multi-scale feature representation.

As illustrated in **Figure 7**, the C3k2_EMA module is proposed by integrating the Efficient Multi-scale Attention (EMA) mechanism into the C3k2 block. EMA significantly bolsters feature representation through cross-space learning and channel dimension reorganization, delivering performance gains with minimal computational overhead.

Specifically, the input feature map is routed through three parallel branches. Two of these utilize 1 × 1 convolutions to model cross-channel dependencies, one extracts linear channel combinations, while the other applies non-linear transformations to enrich channel representation. The third branch employs a 3 × 3 convolution to capture local spatial context and extract adjacent semantic features via an expanded receptive field. Subsequently, the outputs from these three branches are aggregated via element-wise addition to integrate global dependencies with local details. The fused representation is then normalized using a Sigmoid function to generate an attention map, which is applied to the original input via point-wise multiplication to accentuate salient features and suppress background noise.

Unlike conventional attention mechanisms, EMA leverages a parallel multi-scale architecture to aggregate pixel-level contextual information. This design enables the network to adaptively focus on fine-grained floral details, such as petal textures and stamen morphology. Such dynamic weight modulation is crucial for distinguishing target flowers from low-contrast green foliage, mitigating false positives in cluttered backgrounds. By embedding this lightweight module into the neck network, the model substantially improves its spatial sensitivity to occluded targets, ensuring robust perception in unstructured agricultural environments.

#### 2.3.3 PSConv convolution module

**Figure 8:**
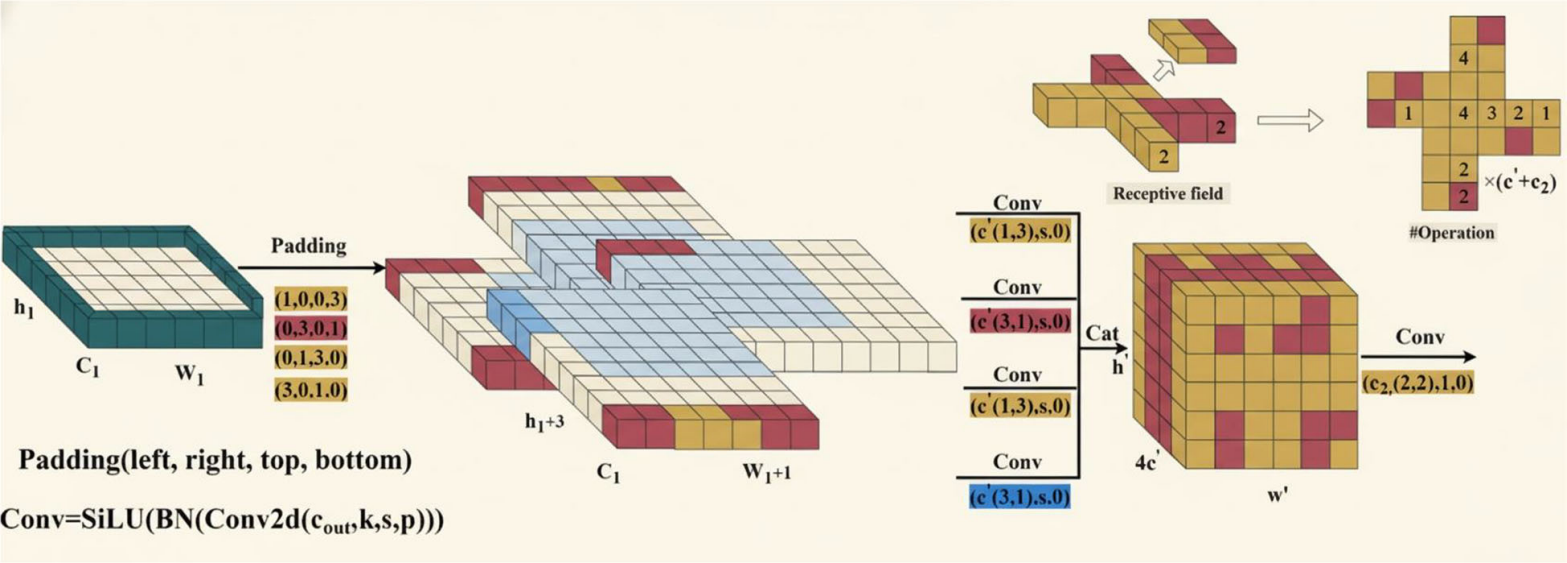
Structure of PSConv convolution module.

To mitigate the inherent information loss associated with standard strided convolutions, we introduce the Pinwheel-shaped Convolution (PSConv) module to replace traditional downsampling layers, as illustrated in **Figure 8**. While conventional downsampling operations effectively reduce spatial dimensions, they suffer from severe detail degradation. This compromises the receptive field coverage and ultimately hinders the accurate detection of minute defective targets. To circumvent this limitation while simultaneously optimizing computational efficiency, expanding the receptive field, and capturing multi-scale features, we replace the downsampling convolution in the original CCFF module with the novel, plug-and-play PSConv. The CCFF module is a core multi-scale feature fusion component in our network architecture, designed to aggregate complementary information from different levels of feature maps and enhance cross-channel feature interaction.

PSConv employs a spatially asymmetric padding strategy, applying distinct horizontal and vertical kernels to specific regions of the feature map. Initially, the input is partitioned into four parallel branches with specific padding configurations (*P*(1,0,0,3), *P*(0,3,0,1), *P*(0,1,3,0), *P*(3,0,1,0)). Each branch is subsequently processed by either a 1×3 or 3×1 convolutional kernel. The proposed computational equation is presented in Equation 5:

(5) $$\begin{array}{l} X_{1}^{\left(h^{\prime },w^{\prime },c^{\prime }\right)}=\text{SiLU}\left(\text{BN}\left(X^{\left(h_{1},w_{1},c\right)}⊗W_{1}^{\left(1,3,c^{\prime }\right)}\right)\right),\\ X_{2}^{\left(h^{\prime },w^{\prime },c^{\prime }\right)}=\text{SiLU}\left(\text{BN}\left(X^{\left(h_{1},w_{1},c\right)}⊗W_{2}^{\left(3,1,c^{\prime }\right)}\right)\right),\\ X_{3}^{\left(h^{\prime },w^{\prime },c^{\prime }\right)}=\text{SiLU}\left(\text{BN}\left(X^{\left(h_{1},w_{1},c\right)}⊗W_{3}^{\left(1,3,c^{\prime }\right)}\right)\right),\\ X_{4}^{\left(h^{\prime },w^{\prime },c^{\prime }\right)}=\text{SiLU}\left(\text{BN}\left(X^{\left(h_{1},w_{1},c\right)}⊗W_{4}^{\left(3,1,c^{\prime }\right)}\right)\right), \end{array}$$

The outputs from these four branches are then concatenated (Cat) to construct an aggregated feature map *X*′. This intermediate representation is further processed by a 2 × 2 convolution without padding, yielding the final output *Y*. The derived calculation formula is illustrated in Equation 6:

(6) $$\begin{array}{l} h^{\prime }=\frac{h_{1}}{s}+1,\;w^{\prime }=\frac{w_{1}}{s}+1,\;c^{\prime }=\frac{c_{2}}{4},\\ {X^{\prime }}^{\left(h^{\prime },w^{\prime },4c^{\prime }\right)}=\text{Cat}\left(X_{1}^{\left(h^{\prime },w^{\prime },c^{\prime }\right)},\ldots ,X_{4}^{\left(h^{\prime },w^{\prime },c^{\prime }\right)}\right),\\ h_{2}=h^{\prime }-1=\frac{h_{1}}{s},\;w_{2}=w^{\prime }-1=\frac{w_{1}}{s},\\ Y^{\left(h_{2},w_{2},c_{2}\right)}=\text{SiLU}\left(\text{BN}\left({X^{\prime }}^{\left(h^{\prime },w^{\prime },4c^{\prime }\right)}⊗W^{\left(2,2,c_{2}\right)}\right)\right). \end{array}$$

By leveraging the principles of group convolution, PSConv effectively expands the receptive field while curtailing the overall parameter burden. Specifically, the parameter count of a standard convolution (*Comparams* = *c*_2_×*c*_1_×*k*×*k*) is compared with that of PSConv. In this derivation, we explicitly assume that the number of input and output channels are equal (*c*_2_ = *c*_1_), which is the standard configuration when PSConv is used to replace downsampling layers in the proposed network architecture. The specific mathematical formulation is shown in Equation 7:

(7) $$PSConv_{params}=4\times \left(c_{2}/4\times c_{1}\times 3\times 1\right)+4c_{2}c_{1}=7c_{2}c_{1}=7c_{1}^{2},$$

Furthermore, qualitative visualizations demonstrate that PSConv accurately delineates target boundaries, suppresses irrelevant background noise, and facilitates the precise localization and morphological identification of small targets. The integration of PSConv not only bolsters the extraction of low-level fine-grained features but also accurately models the spatial Gaussian distribution characteristics of small objects, thereby elevating overall feature extraction precision. Crucially, as a plug-and-play component, PSConv significantly improves small object localization without imposing heavy computational overhead, making it highly suitable for resource-constrained edge deployment in agricultural robotic vision systems.

#### 2.3.4 SimAM lightweight parameter-free attention mechanism

**Figure 9:**
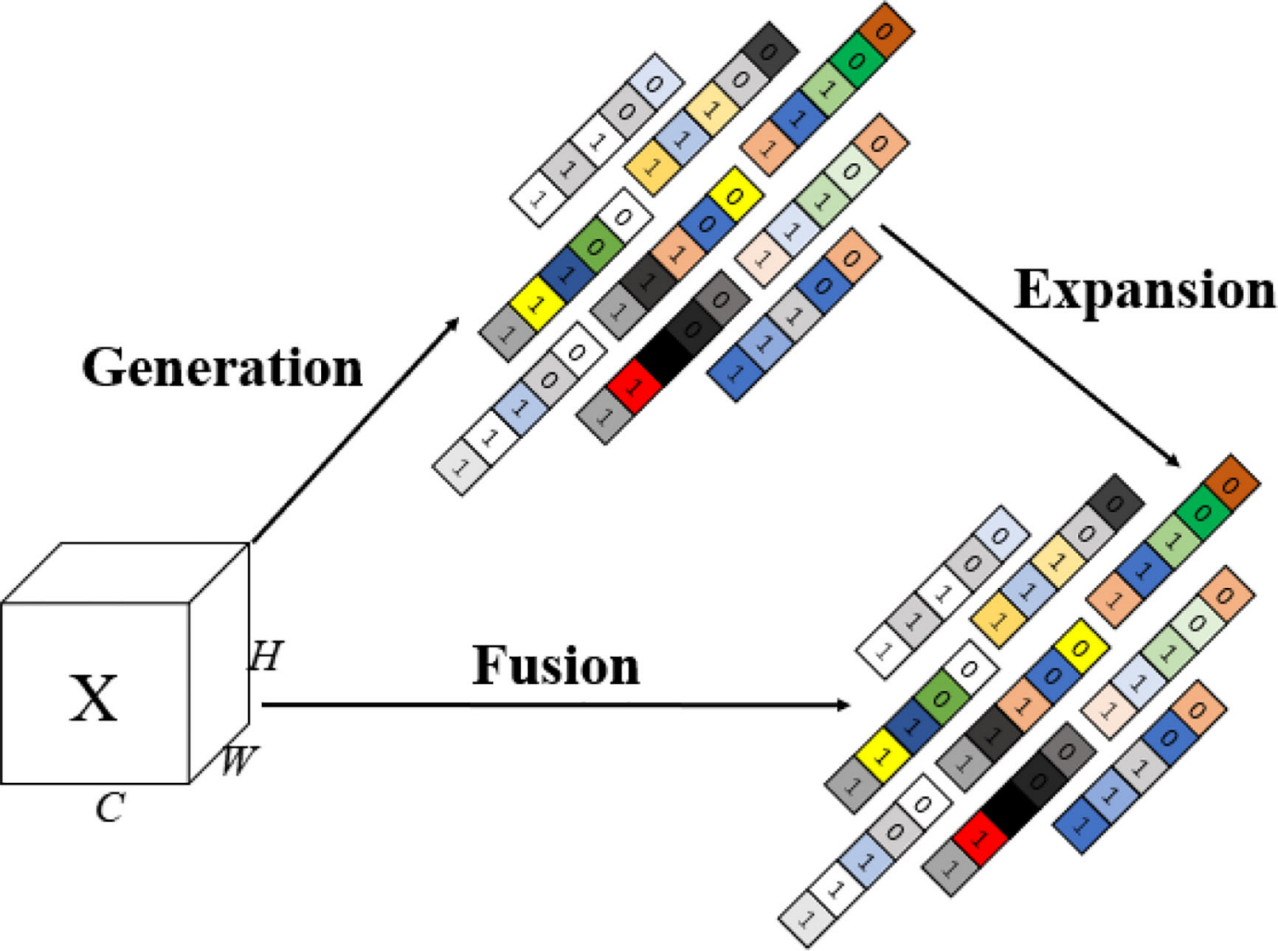
SimAM attention mechanism structure.

Conventional attention mechanisms typically decouple the spatial and channel dimensions, restricting three-dimensional feature synergy. To address this limitation, this study introduces the Simple Attention Module (SimAM) as illustrated in **Figure 9**. As a completely parameter-free mechanism, SimAM jointly models the spatial and channel dimensions. It adaptively evaluates the attention weight for each spatial position, guiding the network to prioritize critical semantic features related to chili flower posture via a mathematically defined energy function. For a target neuron within a specific feature map channel, the energy function for a target neuron on a feature map channel is formulated as:

(8) $$e_{t}^{*}=\frac{4\left({\hat{\sigma }}^{2}+\lambda \right)}{{\left(t-\hat{\mu }\right)}^{2}+2{\hat{\sigma }}^{2}+2\lambda }$$

where the mean $\hat{\mu }$ and variance ${\hat{\sigma }}^{2}$ of all *M* neurons on the channel are calculated as:

(9) $$\hat{\mu }=\frac{1}{M}\sum_{i=1}^{M}x_{i}$$

(10) $${\hat{\sigma }}^{2}=\frac{1}{M}\sum_{i=1}^{M}{\left(x_{i}-\hat{\mu }\right)}^{2}$$

In Equations 8–10, *λ* represents a regularization coefficient to ensure numerical stability, and *M* is the total number of spatial neurons per channel. The calculated energy value inversely reflects the linear separability between the target neuron and its adjacent neighbors. Specifically, a lower energy value indicates a higher degree of distinctiveness, signifying that the neuron encodes highly salient geometric and textural features crucial for chili flower pose estimation, including petal orientation and stamen configuration.

To amplify these posture-related features for subsequent convolutional layers, the energy values are inverted and normalized via a Sigmoid activation function. The resulting 3D attention weights are then fused with the original feature map through element-wise multiplication to yield the refined output in Equation 11:

(11) $$X^{\prime }=\text{Sigmoid}\left(\frac{1}{E}\right)⊙X$$

where *X* is the original input feature map, *X*′ denotes the refined output feature map, *E* represents the set of all energy values $e_{t}^{*}$ across the feature map, and ⊙ denotes the element-wise multiplication operation.

By optimizing this energy function, SimAM deduces full 3D attention weights without introducing any learnable parameters. In the context of chili flower detection, targets are often affected by subtle posture variations, severe foliage occlusion, and background clutter from green leaves and soil. To tackle these unstructured environmental challenges, we strategically embed the SimAM module into the YOLOv8n backbone immediately preceding the SPPF layer. This integration empowers the network to adaptively highlight fine-grained structural features, particularly petal edges and stamen morphology, while simultaneously suppressing background noise. Consequently, the model achieves superior precision in posture estimation and demonstrates enhanced robustness against feature drift induced by dynamic illumination and overlapping canopies. Furthermore, the parameter-free design of SimAM preserves the ultra-low computational complexity of the baseline model, facilitating highly efficient and real-time deployment on resource-constrained agricultural edge devices like robotic pollination platforms.

### 2.4 Test environment and evaluation metrics

#### 2.4.1 Test environment

This research was conducted on the Windows 10 operating system using the PyCharm Community Edition 2024.2.3 development environment, Python 3.9.20, and the PyTorch 2.3.0 deep learning framework for both model training and testing. The hardware configuration included an Intel^®^ Core™ i7-13700KF processor and an NVIDIA GeForce RTX 3090 graphics card with 24GB of VRAM, ensuring adequate computational power for handling complex deep learning tasks. To maintain consistency and enable fair comparison of experimental results, all models were trained and tested under an identical parameter configuration. Detailed training parameters are provided in **Table 1**. In particular, a constant learning rate of 0.01 was adopted throughout the training process without any decay or scheduling strategy, so the initial and final learning rates remain identical at 0.01.

**Table 1:** Training parameters.

Training parameter	Value or type
Input image size	640 × 640 × 3
Optimizer	SGD
Optimizer momentum	0.937
Optimizer weight decay rate	0.0005
Batch size	16
nc	5
Epoch	300

#### 2.4.2 Model evaluation metrics

This study employs *P*, *R*, *mAP*, the number of parameters (Parameters), and frames per second (FPS) as the primary evaluation metrics for assessing detection performance in chili flower pose recognition. *P* measures the proportion of correctly identified positive samples among all samples predicted as positive, while *R* indicates the proportion of positive samples accurately detected among all actual positives. The *mAP*_50_ is calculated based on detection results with an intersection over union (IoU) threshold of 0.5. The calculation of the mean average precision (*mAP*_50−95_) is based on the intersection over union. The test results are measured when the IoU threshold is set at 0.5-0.95, ensuring a balanced assessment of accuracy and generalization. The number of parameters reflects the model’s structural complexity, whereas FPS evaluates its real-time processing capability and computational efficiency. The formulas for each metric are provided in Equations 12–15.

(12) $$P=\frac{C_{TP}}{C_{TP}+C_{FP}}\times 100\%$$

(13) $$R=\frac{C_{TP}}{C_{TP}+C_{FN}}\times 100\%$$

(14) $$AP=\int_{0}^{1}P\left(R\right)dR$$

(15) $$mAP=\frac{\sum_{i=1}^{n}AP_{i}}{n}$$

In the formula, *C_TP_*refers to the number of instances in which the model correctly predicts the actual posture of a given chili flower type as its corresponding posture. *C_FP_*represents the number of instances where the model incorrectly classifies a posture from another chili flower type as belonging to this type. *C_FN_*denotes the number of instances where the model fails to recognize the correct posture of a chili flower type and instead classifies it as belonging to a different type. Furthermore, *AP* indicates the model’s overall ability to accurately identify and distinguish the postures across different chili flower types.

## 3 Results and discussion

To evaluate the effectiveness of the CFPR-YOLO model in recognizing chili flower postures, this study conducted experimental assessments from four perspectives: ablation studies, comparative experiments, visualization of detection results, and heat map analysis. These evaluations provided a comprehensive examination of the model’s performance with respect to detection accuracy, computational efficiency, and deployment capability.

### 3.1 Ablation experiment

Ablation experiments serve as a vital validation technique in the optimization of deep learning models, as they systematically remove or alter specific components to assess each module’s contribution to overall performance. In this study, a detailed ablation analysis was carried out on the proposed CFPRYOLO model, examining both the independent and combined effects of five core improvement modules: A (EfficientFormerV2 backbone network), B (C3k2_EMA feature enhancement module), C (PSConv convolution module), and D (SimAM attention mechanism). The experiments were conducted using the YOLOv11n model as the baseline and were quantitatively evaluated across multiple dimensions, including detection accuracy (P, R, mAP), computational efficiency (number of parameters, FLOPs), and real-time performance (FPS), along with other critical indicators. As summarized in **Table 2**, the ablation study not only confirmed the technical effectiveness of each module but also provided a solid scientific foundation for optimizing the overall model architecture.

**Table 2:** CFPR-YOLO ablation experiment results.

Model	Improvement module				Evaluation index				
	A	B	C	D	P/%	R/%	mAP50/%	mAP50-95/%	Params (M)
YOLOv11n	–	–	–	–	92.8	85.7	90.7	63.4	**2.58**
YOLOv11n	✓	–	–	–	92.7	86.2	91.1	64.0	4.84
YOLOv11n	–	✓	–	–	93.1	84.3	90.5	62.8	2.59
YOLOv11n	–	–	✓	–	92.2	85.5	90.4	62.9	2.46
YOLOv11n	–	–	–	✓	92.3	85.0	90.5	62.9	2.58
YOLOv11n	✓	✓	–	–	91.4	86.6	91.5	64.7	7.41
YOLOv11n	✓	–	✓	–	93.4	84.6	91.5	65.0	7.29
YOLOv11n	✓	–	–	✓	92.6	85.4	91.1	64.6	7.36
YOLOv11n	✓	✓	✓	–	93.7	84.9	91.4	64.9	7.26
YOLOv11n	✓	✓	–	✓	92.4	86.2	91.2	64.7	7.36
YOLOv11n	✓	–	✓	✓	92.2	85.9	90.9	64.5	7.88
YOLOv11n	✓	✓	✓	✓	92.6	**86.8**	**92.1**	**64.9**	7.26

✓ indicates using this module, and “–” means not using this module.

Bold values denote the optimal performance under each evaluation metric.

Ablation experiments verified the effectiveness of the hierarchical optimization strategy. The baseline YOLOv11n achieved an mAP50 of 90.7%. The integration of individual and combined modules led to a progressive enhancement in detection performance. Specifically, replacing the backbone with EfficientFormerV2 (Module A) enhanced global feature extraction capabilities, elevating mAP50 to 91.1% and Recall to 86.2%. The subsequent integration of the C3k2_EMA module (Module B) further pushed mAP50 to 91.5% by strengthening multi-scale semantic consistency through cross-dimensional attention. Although this step induced minor fluctuations in Precision, the introduction of the PSConv module (Module C) effectively resolved this by optimizing spatial feature sampling topology, raising Precision to a peak of 93.7% in the A+B+C configuration while maintaining a compact parameter count of 7.26M. Finally, the incorporation of the parameter-free SimAM attention mechanism (Module D) refined the detection head by evaluating neuronal energy contributions, achieving the best comprehensive performance with an mAP50 of 92.1% and a high-threshold mAP50–95 of 64.9%. The cumulative gains of 1.4 and 1.5 percentage points in mAP50 and mAP50-95, respectively, over the baseline confirm that the synergistic integration of these modules successfully balances high-precision localization of tiny targets with robust feature recognition in unstructured agricultural environments.

To further elucidate the internal mechanism, the analysis is conducted from three dimensions: First, the transition from local texture capture to global semantic understanding enabled by Module A and B suppresses background interference from similar-textured foliage. Second, the spatial reconstruction provided by PSConv (Module C) models the delicate geometric correlations between stamens and petals, which is crucial for pose estimation under severe occlusion. Third, the SimAM (Module D) serves as a refined filter at the detection head, significantly mitigating bounding box offsets under complex lighting conditions. These combined improvements provide reliable kinematic guidance for the high-precision operations of pollination robots.

### 3.2 Comparative experiments

#### 3.2.1 Comparison test of different backbone networks replacement

To systematically examine the impact of different backbone networks on the performance of the chili flower pose estimation model, three comparative experiments were conducted. In these experiments, the original YOLOv11n backbone was respectively replaced with MobileNetV4, StarNet, and EfficientFormerV2, and their performances were evaluated against the baseline model. The corresponding experimental results are summarized in **Table 3**.

**Table 3:** Comparison of model performance for adding different backbone networks.

The replaced backbone network	P/%	R/%	mAP50/%	mAP50-95/%
Initial backbone	**92.8**	85.7	90.7	63.4
MobileNetV4	90.3	84.7	90.0	61.7
StarNet	90.8	83.4	90.2	61.6
EfficientFormerV2	92.7	**86.2**	**91.1**	**64.0**

Bold values denote the optimal performance under each evaluation metric.

To determine the optimal feature extractor for chili flower pose estimation, we compared EfficientFormerV2 against MobileNetV4 and StarNet. Experimental results indicate that EfficientFormerV2 provided the most balanced performance across all key metrics. It achieved an mAP50 of 91.1% and Recall of 86.2%, surpassing the baseline YOLOv11n by 0.4 and 0.5 percentage points, respectively. More importantly, it demonstrated a significant advantage in the mAP50–95 metric, outperforming MobileNetV4 and StarNet by over 2.0 percentage points. This superiority stems from its hybrid Transformer-CNN architecture, which effectively captures both global context information and local detail features. This capability is critical for distinguishing subtle pose variations of small targets in complex field environments, rendering EfficientFormerV2 the ideal backbone for our framework.

#### 3.2.2 Comparative experiments incorporating different attention mechanisms into the C3k2 module

To systematically investigate the impact of different attention mechanisms within the C3k2 module on the performance of the chili flower pose estimation model, three comparative experiments were designed. In these experiments, the EfficientFormerV2 backbone was retained, while the MLCA, Star CAA, and EMA attention mechanisms were individually incorporated into the C3k2 module. Their performances were then compared against the baseline model. The corresponding experimental results are summarized in **Table 4**.

**Table 4:** Comparison of model performance for adding different attention mechanisms incorporated into the C3k2 module.

Attention mechanism in C3k2	P/%	R/%	mAP50/%	mAP50-95/%
YOLOv11n+A	92.7	86.2	91.1	64.0
MLCA	**93.0**	85.9	91.2	64.6
Star CAA	91.9	86.0	90.9	**64.8**
EMA	91.4	**86.6**	**91.5**	64.7

Bold values denote the optimal performance under each evaluation metric.

Integrating the EMA attention mechanism into the C3k2 module significantly bolstered the model’s ability to localize small targets. Among the tested attention mechanisms, EMA secured the highest mAP50 of 91.5% and Recall of 86.6%, outperforming MLCA and Star CAA. Although a slight compromise in Precision was observed, the substantial improvement in Recall is paramount for agricultural applications, where missed detections are more detrimental than false positives. The EMA mechanism effectively aggregates multi-scale features, ensuring that diminutive floral cues are not lost during feature fusion. This robust performance in maintaining high Recall confirms C3k2_EMA as the optimal choice for detecting densely distributed chili flowers under occlusion.

#### 3.2.3 Replace different convolution comparison experiments

To systematically investigate the impact of different convolutional modules on the performance of the chili flower pose estimation model, three comparative experiments were conducted. In these experiments, the EfficientFormerV2 backbone and the EMA attention mechanism were retained within the C3k2 module, while alternative convolutional modules, LDConv, Adown, and PSConv, were individually introduced to replace the standard convolution in the neck network. Their performances were then evaluated against the original model. The corresponding experimental results are summarized in **Table 5**.

**Table 5:** Comparison of model performance with replacement of different convolution modules.

Convolution module	P/%	R/%	mAP50/%	mAP50-95/%
YOLOv11n+A+B	91.4	86.6	91.5	64.7
LDConv	93.0	86.1	91.4	64.5
Adown	91.6	**86.8**	91.3	64.6
PSConv	**93.7**	84.9	**91.4**	**64.9**

Bold values denote the optimal performance under each evaluation metric.

Replacing standard convolution with PSConv proved highly effective in preserving spatial details during downsampling. PSConv achieved a peak Precision of 93.7% and an mAP50–95 of 64.9%, surpassing LDConv and Adown modules. By leveraging multi-scale kernels, PSConv simultaneously captures features from different receptive fields, which is vital for identifying chili flowers of varying sizes and growth stages. While this modification led to a marginal drop in Recall compared to Adown, the significant gains in Precision and overall localization accuracy offer a more pragmatic trade-off for precise pose estimation. Consequently, PSConv was selected to minimize feature information loss while reducing computational redundancy.

#### 3.2.4 Add comparative experiments of different attention mechanisms

To systematically examine the impact of different attention mechanisms on the performance of the chili flower pose estimation model, this study conducted two sets of comparative experiments. In these experiments, the EfficientFormerV2 backbone was adopted, the EMA attention mechanism was integrated into the C3k2 module, and the PSConv convolution module was substituted into the network. Subsequently, the SimAM and ECA attention mechanisms were individually introduced before the three output detection heads, and their performances were compared with that of the original model. The corresponding experimental results are summarized in **Table 6**.

**Table 6:** Comparison of model performance for replacing different detection head modules.

Detection head module	P/%	R/%	mAP50/%	mAP50-95/%
YOLOv11n+A+B+C	**93.7**	84.9	91.4	64.9
ECA	92.3	85.4	90.9	64.5
SimAM	92.6	**86.8**	**92.1**	**64.9**

Bold values denote the optimal performance under each evaluation metric.

The introduction of the parameter-free SimAM attention before the detection head further refined the model’s sensitivity to key floral regions. SimAM increased Recall to 86.8%, a 1.9 percentage point improvement over the configuration without head attention. It outperformed the ECA mechanism in both Recall and mAP50, demonstrating superior capability in suppressing background noise. Although Precision decreased slightly, the overall enhancement in detection completeness and stability across different IoU thresholds validates the value of SimAM. Its parameter-free nature ensures these gains are achieved with negligible computational overhead, making it highly suitable for real-time edge deployment.

#### 3.2.5 Comparison experiments among different models

To comprehensively evaluate the overall performance of the improved CFPR-YOLO model in chili pepper posture recognition, this study conducted comparative experiments with several mainstream object detection algorithms, including FasterRCNN, EfficientDet, RTDETR-r18, YOLOv5n, YOLOv7-tiny, YOLOv8n, YOLOv10n, and YOLOv11n. All models were trained and tested on the same dataset under identical hardware conditions, with parameter configurations strictly controlled to ensure fairness and objectivity. The comparative results across different models are summarized in **Table 7**.

**Table 7:** Comparison of training results of different detection algorithms.

Models	P/%	R/%	mAP50/%	mAP50-95/%	Parameters (M)	FPS (f·s^−1^)
FasterRCNN	60.8	80.5	77.9	60.1	108.26	12.6
EfficientDet	91.9	70.6	79.6	61.2	15.03	22.3
RTDETR-r18	91.2	**87.1**	89.2	64.3	19.88	74.8
YOLOv7-tiny	91.8	85.6	89.2	61.5	6.02	34.9
YOLOv8n	92.4	85.1	90.3	62.8	3.01	**183.2**
YOLOv10n	91.2	86.3	90.1	62.8	2.70	131.0
YOLOv11n	**92.8**	85.7	90.7	63.4	2.58	155.0
CFPR-YOLO	92.6	86.8	**92.1**	**64.9**	7.26	35.1

Bold values denote the optimal performance under each evaluation metric.

Comparative experimental results across different detection models are summarized in **Table 7**. The proposed CFPR-YOLO achieves competitive detection performance under the tested conditions, with consistently strong results across multiple evaluation metrics.

From the perspective of precision and recall, CFPR-YOLO maintains a balanced detection behavior, indicating its ability to handle both accurate localization and comprehensive target coverage. In contrast, Faster R-CNN exhibits relatively limited precision and only moderate recall, suggesting difficulties in handling small and densely distributed targets. EfficientDet shows relatively strong precision but lower recall, which implies that some targets may be missed in complex scenarios. Compared with these methods, CFPR-YOLO provides a more stable balance between precision and recall.

In terms of localization accuracy, CFPR-YOLO achieves the best overall performance among the compared methods. It demonstrates consistent improvements over RTDETR-r18 and YOLOv8n, particularly under stricter evaluation criteria, reflecting more reliable localization in challenging conditions.

Regarding model complexity, CFPR-YOLO introduces a moderate increase in parameter scale compared with lightweight models, while remaining significantly more compact than traditional detection frameworks. This indicates that the performance improvement is primarily achieved through effective architectural design rather than excessive parameter expansion.

In terms of inference efficiency, CFPR-YOLO maintains real-time performance on the tested platform. Although it is not as fast as highly optimized lightweight models, it still achieves a reasonable balance between speed and accuracy, while demonstrating clear efficiency advantages over heavier detection models.

Overall, the results indicate that CFPR-YOLO achieves a well-balanced trade-off among detection accuracy, robustness, and computational efficiency within the tested dataset and hardware environment.

**Figure 10:**
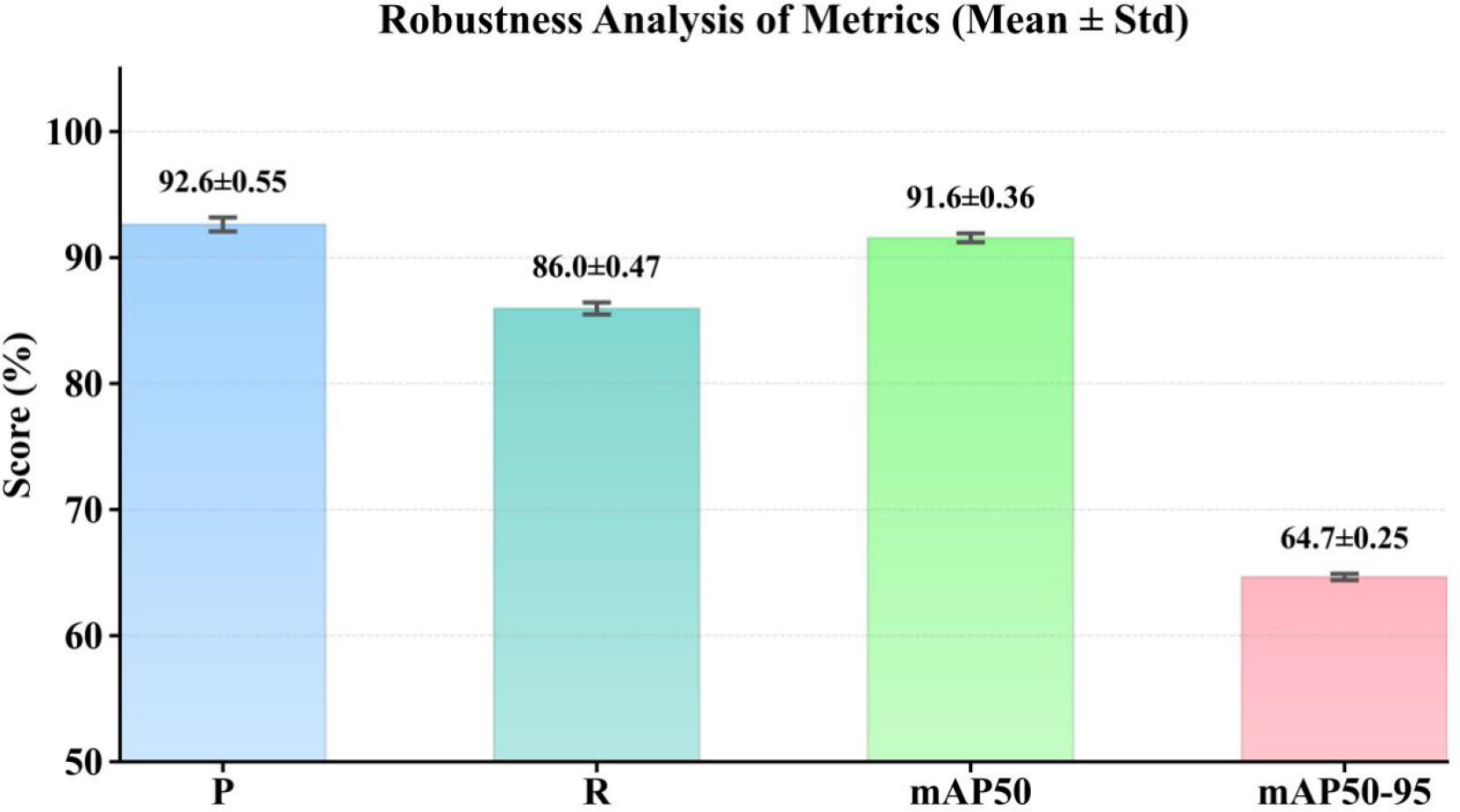
Comparative analysis of variance in repeated experiments.

To eliminate the interference of stochastic parameter initialization during deep learning training on experimental conclusions and to comprehensively evaluate the robustness and reproducibility of the CFPRYOLO model, this study adopted a five-fold repeated experiment strategy. While maintaining consistent hyperparameters, five random seeds widely recognized in the deep learning community (Default, 0, 1, 42, 3407) were selected for independent training. **Table 8**; **Figure 10** present the performance metrics and their standard deviations (Std) under different initialization conditions.

**Table 8:** Repeated experiments and analysis of STD.

Seed	Repeated experiments				StD			
	P	R	mAP50	mAP50-95	P	R	mAP50	mAP50-95
Default	92.6	86.8	92.1	64.9				
0	93.3	85.7	91.5	64.9				
1	91.8	85.8	91.1	64.3	0.55	0.47	0.36	0.25
42	92.5	85.8	91.5	64.6				
3407	92.9	85.7	91.6	64.6				

Statistical results demonstrate that the model exhibits exceptional stability in comprehensive performance. The standard deviations for mAP50 and mAP50-95, which serve as core metrics for assessing detection quality, were controlled at extremely low levels of 0.36 and 0.25, respectively. This indicates that despite the minor perturbations introduced by random initialization, the model consistently converges to the vicinity of a similar global optimum across different conditions. This stability is attributed to the enhanced feature extraction capabilities provided by the EfficientFormerV2 backbone and the SimAM attention mechanism, thereby validating the inherent stability of the architectural design.

In contrast, Precision and Recall exhibited marginal fluctuations and demonstrated a discernible trade-off relationship. Specifically, the Seed 0 group displayed a “high precision, low recall” characteristic, achieving a Precision of 93.3% and a Recall of 85.7%, whereas the Default group performed better in Recall, reaching 86.8%. The primary reasons for these inter-group variations are twofold: 1) Non-convexity of the optimization landscape: Different random seeds alter the initial distribution of detection head weights, causing minor shifts in the classification decision boundary upon convergence, which consequently induces a trade-off between Precision and Recall. 2) Stochasticity of data augmentation: The random stitching processes in Mosaic and Mixup augmentations within the YOLO training framework are seed-controlled. The Seed 1 group, with an mAP50–95 of 64.3%, performed slightly below the average, likely due to exposure to a higher proportion of difficult synthetic samples during the early training stages, leading the optimizer into a slightly inferior local minimum. Nevertheless, even under the least favorable random seed configuration, the model’s performance remained significantly superior to the baseline model. Furthermore, the average mAP50–95 across the five experiments stabilized at approximately 64.7%. These findings provide compelling evidence that the effectiveness of the proposed method stems from the superiority of the architectural design rather than accidental gains from specific parameter initializations.

**Figure 11:**
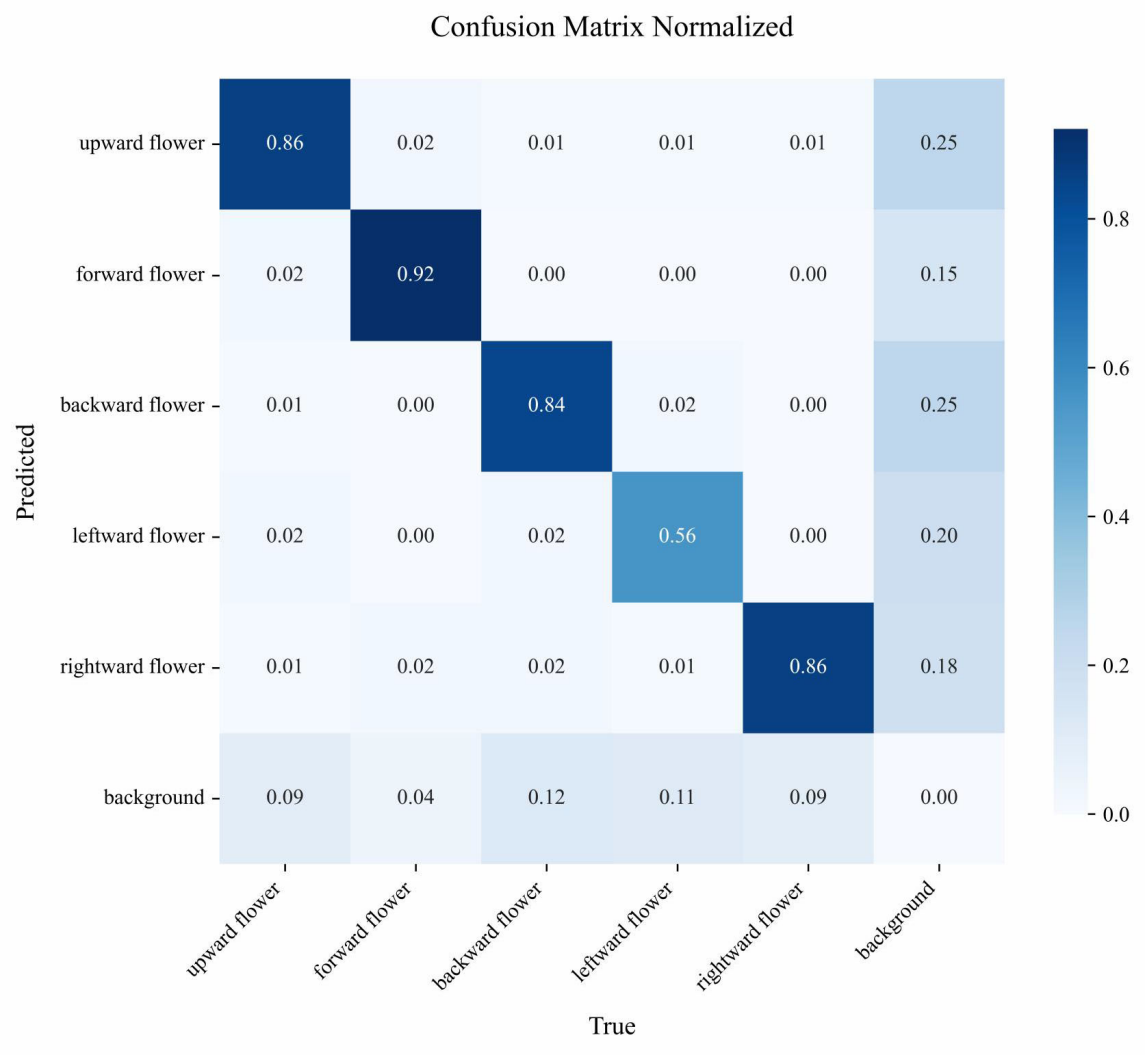
Confusion matrix normalized.

**Figure 11** illustrates the normalized confusion matrix for the model across five chili flower pose categories and the background class. Overall, the model demonstrates exceptional pose decoupling capability and classification robustness. As evidenced by the diagonal elements, the Recall rates for all pose categories are maintained within a high-confidence interval of 0.84 to 0.92. Furthermore, the probability of confusion between different pose categories is negligible (off-diagonal values are generally below 0.02). This indicates that the feature vectors extracted by the model possess strong discriminability, effectively capturing subtle morphological differences such as pistil orientation and petal symmetry. Consequently, the model precisely distinguishes between symmetrical poses like “Leftward” and “Rightward,” providing reliable pose priors for robotic path planning.

Regarding the “Upward” category, which is the core target for robotic pollination operations, the model achieved a correct recognition rate of 0.86. This implies that the vast majority of viable pollination targets can be successfully captured by the visual system. However, it is notable that the False Positive rate for this category against the background class reached 0.25 (upper-right region of the matrix). This phenomenon is primarily attributed to the complex unstructured field environment: specular reflections on upwardgrowing young leaves or unopened buds share high textural and geometric similarities with blooming upward flowers, leading to false alarms. Therefore, in practical pollination strategies, it is recommended to implement a stricter confidence threshold for the “Upward” category to optimize pollination success rates while ensuring operational safety.

In the comparative analysis of other pose categories, the “Forward” class exhibited superior performance, achieving the highest accuracy of 0.92 with a minimal miss rate of 0.04. This is largely due to the full exposure of distinct pistil and petal structures from this perspective. Conversely, the “Backward” class presented the most significant challenge (Accuracy 0.84, Miss Rate 0.12). This is because the dorsal view only captures the calyx and pedicel, whose color features blend heavily with the surrounding stem and foliage background, lacking distinct texture gradients, which results in some targets being misclassified as background. In summary, the model excels in pose classification tasks, and future optimization will focus on further suppressing false positives in complex backgrounds through keypoint estimation.

### 3.3 Comparison of visualization detection results of each model

**Figure 12:**
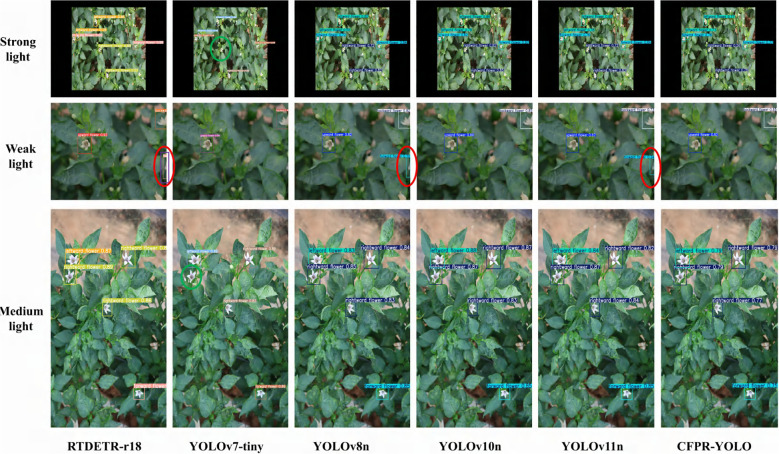
Comparison of the visual detection effect of each model under different lighting conditions.

**Figure 13:**
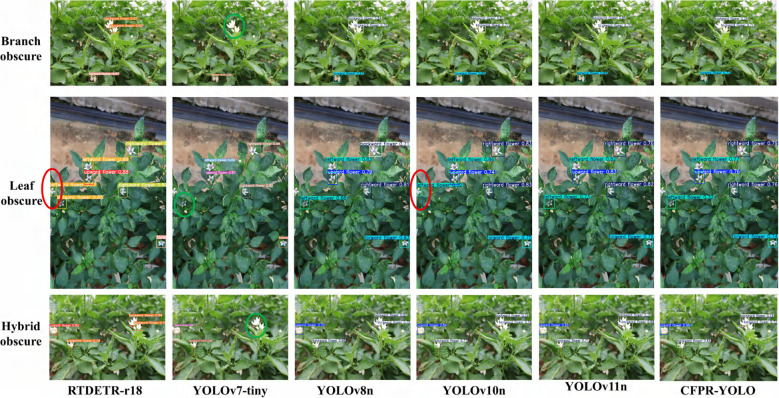
Comparison of the visual detection effect of each model under different occlusion conditions.

The performance of object detection models largely depends on the availability of high-quality datasets. In facility agriculture environments, capturing images of chili flowers is particularly challenging, as the shooting conditions are often affected by factors such as occlusion and uneven lighting. These conditions result in a limited number of original image samples and an imbalanced distribution, which in turn hinder the training effectiveness of the model. To enhance the robustness and generalization ability of the CFPRYOLO model, this study conducts experiments using both the original image dataset and test datasets generated after applying a series of carefully designed data enhancement strategies. The visualization results from the preliminary tests were then compared and analyzed. **Figures 12**, **13** present the comparative detection outcomes under varying lighting and occlusion conditions, respectively.

As shown in **Figure 12**, within natural pollination scenarios, chili flowers are prone to false or missed detections due to fluctuations in lighting, especially under extreme variations in light intensity. The experimental results indicate that the RTDETR-r18 and YOLOv8n models suffer from reduced accuracy in low-light environments, often producing false detections of chili flowers. Similarly, the YOLOv7-tiny model experiences missed detections under both strong and moderate lighting, reflecting its high sensitivity to illumination changes. In contrast, the YOLOv10n model remains unaffected by light variation, showing no missed or false detections, exhibiting stable detection performance under various lighting conditions. However, the YOLOv11n model is significantly impacted by inadequate lighting, leading to false detections in low-light scenarios. By comparison, the CFPR-YOLO model achieves higher effectiveness in mitigating the interference caused by lighting variations. It consistently recognizes chili flowers against complex leaf backgrounds, generating tighter detection bounding boxes with more accurate positioning. These improvements reflect the favorable feature extraction and small-target detection capabilities of the upgraded module.

As illustrated in **Figure 13**, under natural pollination conditions, chili flowers are highly vulnerable to recognition challenges such as incomplete feature identification, partial loss of key attributes, and blurred boundaries caused by different levels of occlusion. These issues often lead to false detections or missed detections, with performance degradation becoming more evident when flowers are heavily obscured. The experimental results reveal that the RTDETR-r18 model is unable to effectively suppress interfering elements in leaf-occlusion scenarios, resulting in frequent false detections. Similarly, in multiple occlusion settings, the YOLOv7-tiny model shows a sharp decline in detection accuracy, accompanied by noticeable instances of missed detections. On the other hand, both YOLOv8n and YOLOv11n demonstrate strong adaptability in diverse occlusion environments, indicating that they possess relatively high robustness against occlusion-related challenges. However, the YOLOv10n model tends to produce false detections under leaf occlusion, suggesting that its robustness weakens when dealing with occlusion conditions involving similar colors and structural patterns.

In contrast, the CFPR-YOLO model exhibits favorable detection performance in addressing the interference caused by occlusion. It consistently achieves accurate identification of chili flowers, even in the presence of complex overlapping structures, while also performing reliable pose estimation. This performance verifies the promising potential of CFPR-YOLO in handling occlusion interference, ensuring precise detection under complex field conditions.

**Figure 14:**
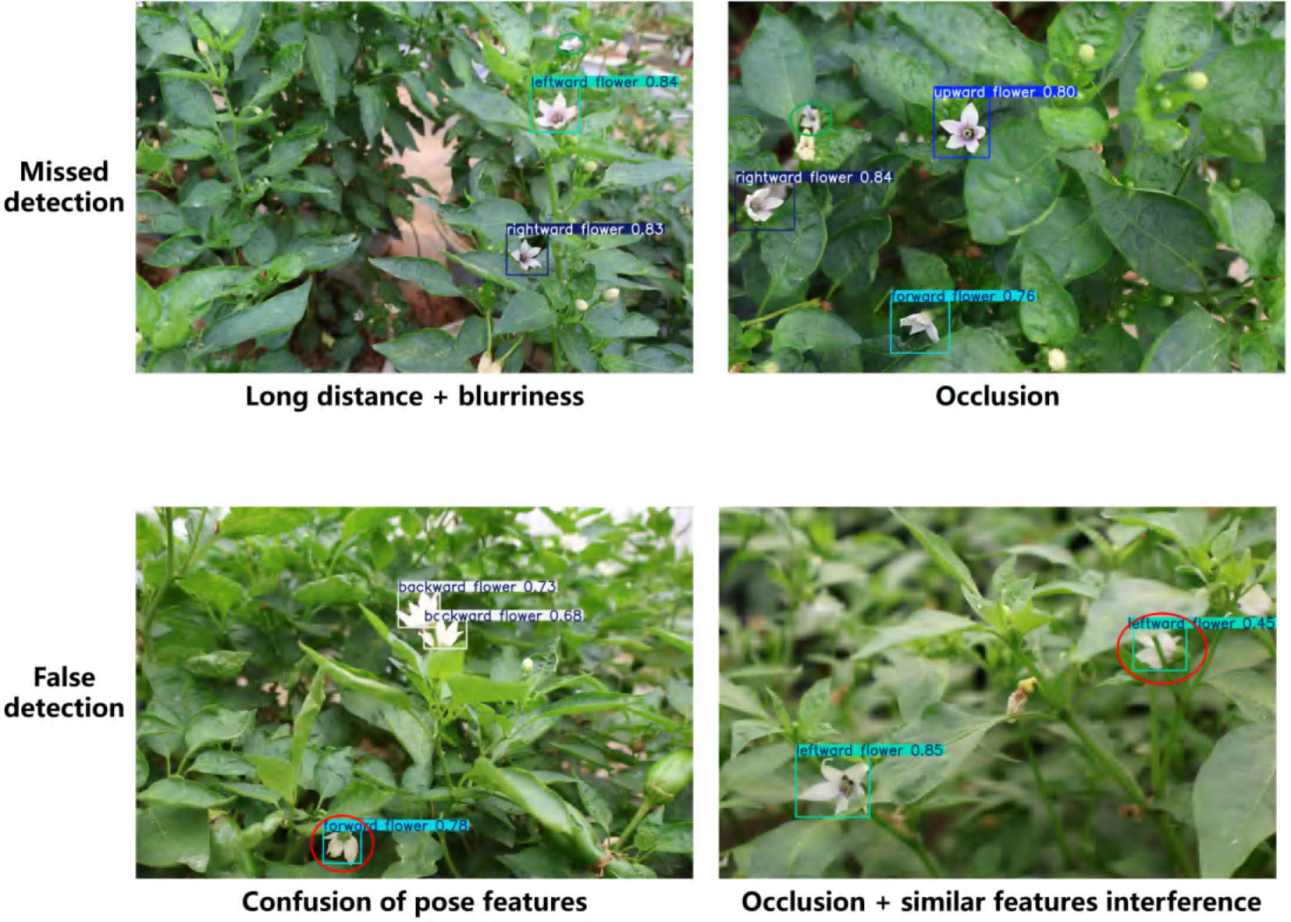
False detection and missed detection analysis of the cfpr-yolo model.

Despite the superior detection performance of the CFPR-YOLO model on the overall test set, a small number of False Positives and False Negatives persist under extremely complex unstructured field conditions, as illustrated in **Figure 14**.

Missed detections (indicated by green circles) primarily occur in scenarios involving blur caused by long-distance shooting and high-density foliage occlusion. In distant and blurred scenes, the chili flower occupies a minimal portion of the image, and edge textures are smoothed. This leads to the loss of key semantic features during the convolutional network’s downsampling process, making it difficult to activate the target region against the background. In occluded scenarios, foreground leaves not only disrupt the geometric integrity of the flower but also block key visual contours that distinguish the flower from the background. Due to insufficient sensitivity to tiny and feature-incomplete targets, these highly concealed flowers are judged as background, resulting in missed detections.

False detections (indicated by red circles) primarily stem from the confusion of pose features and the alteration of the target’s visual topological structure due to occlusion. In the “Confusion of pose features” scenario, the growth orientation of chili flowers often exhibits a continuous spatial transition rather than a strictly discrete distribution. Specifically, for flowers in intermediate poses (e.g., “Left-Upward”), the geometric morphology of their petal unfolding presents significant visual ambiguity when projected onto a 2D plane. Under conditions of intense illumination or indistinct textural details, the visual characteristics of these targets—which simultaneously possess “Leftward” and “Upward” tendencies—become highly susceptible to confusion with “Forward,” “Leftward,” or standard “Upward” flowers. This high degree of inter-class similarity hinders the model’s ability to discern subtle depth and angular deviations, leading to the extraction of erroneous feature vectors. Consequently, flowers actually growing laterally or obliquely are misclassified as “Upward” or “Forward” poses. Furthermore, in the “Occlusion + similar background interference” scenario, physical occlusion fundamentally alters the visible geometry of the target. For instance, a “Backward” flower exposing only partial petals may geometrically resemble the lateral profile of a “Leftward” pose. Compounded by interference from cluttered background textures, this results in misclassification by the model’s classifier within the feature mapping space. These findings indicate that the model continues to face challenges in addressing local feature absence and performing fine-grained pose discrimination.

**Figure 15:**
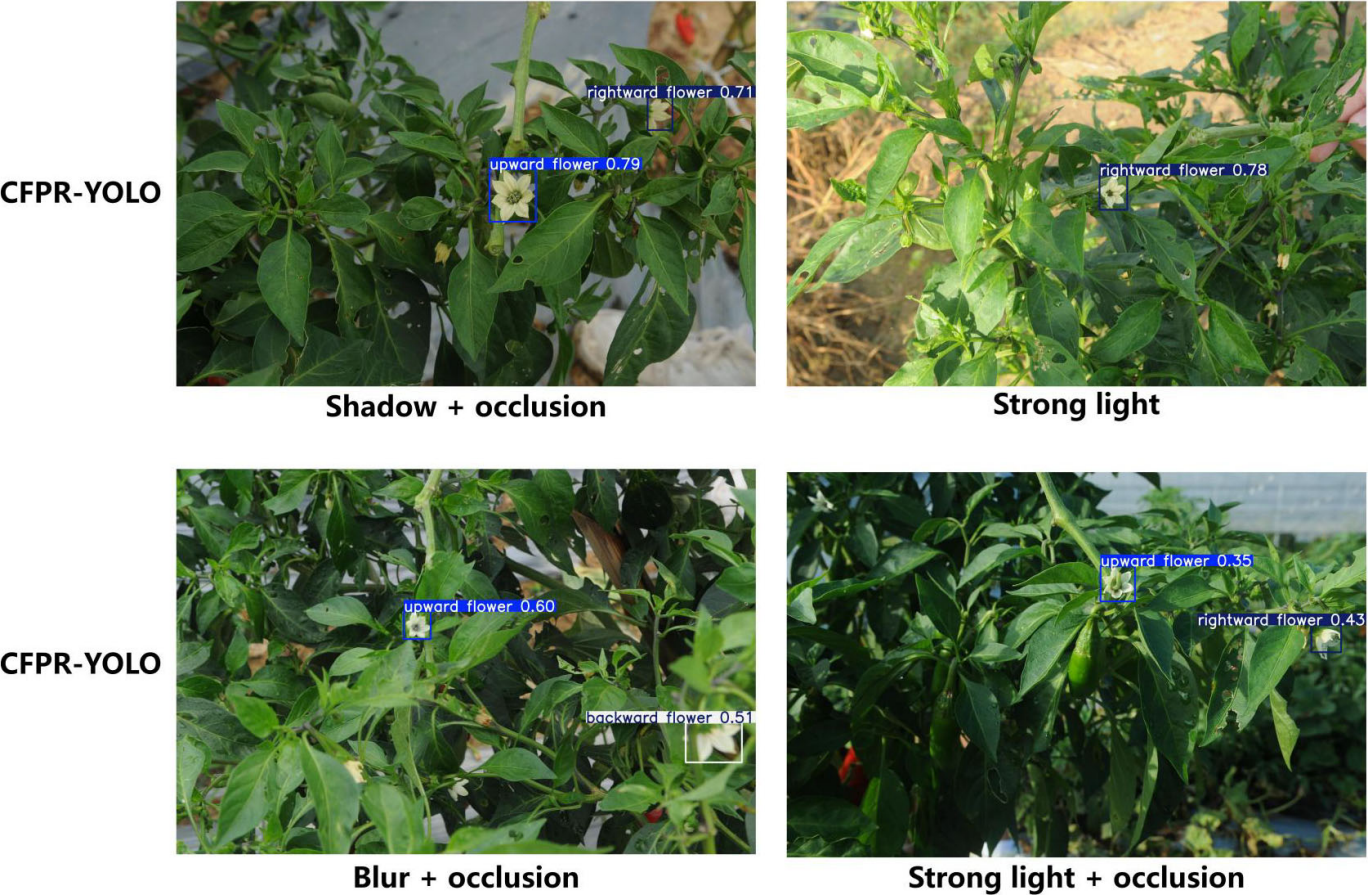
Generalization experiment results of the CFPR-YOLO model.

To evaluate the generalization capability of the model across different chili varieties, this study introduced an independent external dataset from our previous work published in Applied Soft Computing Gao et al. (2023). This external dataset contains 6419 images, covering three typical varieties: Zhangshugang S8, Zunla No. 1, and Changsha Dongshan Glossy Chili Pepper. This dataset covers chili varieties with significant morphological differences, such as Zhangshugang S8, Zunla No. 1, and Changsha Dongshan Glossy Chili Pepper. We employed a random sampling strategy to construct a test library containing an independent subset from this dataset for cross-domain performance validation. As shown in the visual detection results in **Figure 15**, although the test samples differ significantly from the training set in terms of floral morphological structure, growth density, and background data distribution, the proposed model demonstrated favorable adaptability. It successfully achieved precise localization of new variety flowers not involved in training, with high bounding box tightness, indicating acceptable feature transferability and generalization ability under variety changes and environmental variations.

Despite the model’s overall excellence, deep error analysis in complex field scenarios reveals its limitations regarding false and missed detections under extreme conditions.

**Figure 16:**
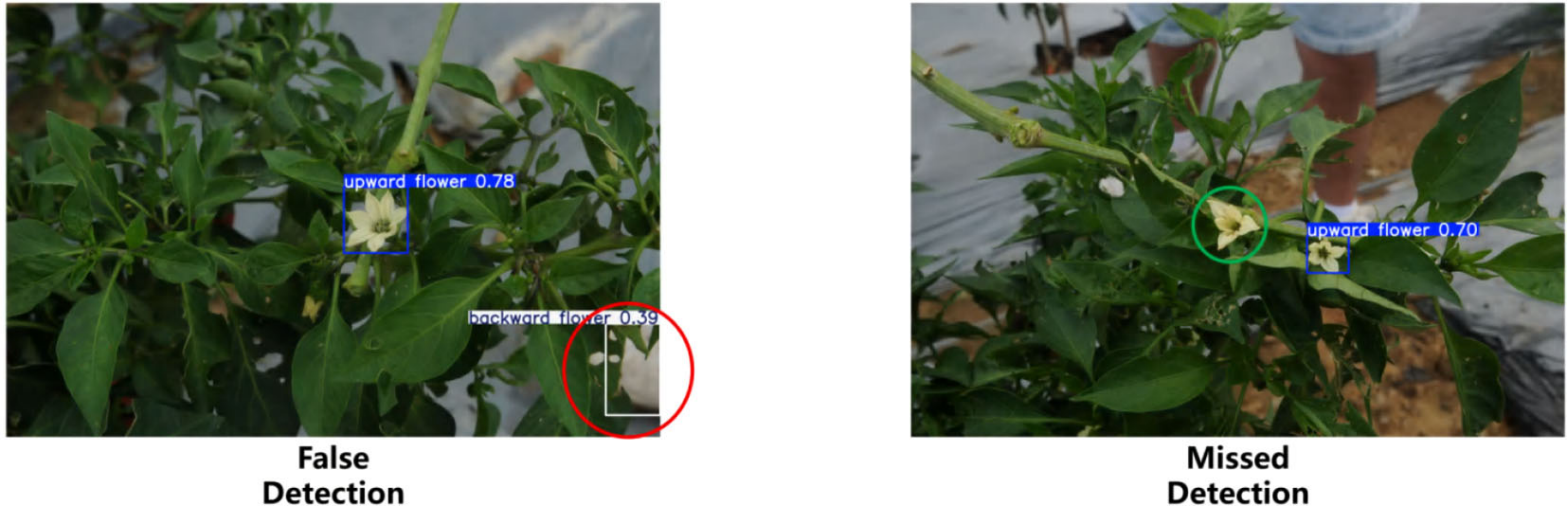
Analysis of false detection and missed detection in the generalization of the cfpr-yolo model.

Firstly, False Positives mainly occur in regions with high-frequency color background interference. As shown in **Figure 16**, highly similar background colors lead to white background features being identified as “Backward” flower poses. These features bear a high color resemblance to the white petals of backwardfacing flowers, easily confusing the model’s judgment and resulting in false detections.

### 3.4 Chili flower heat map detection image comparison

**Figure 17:**
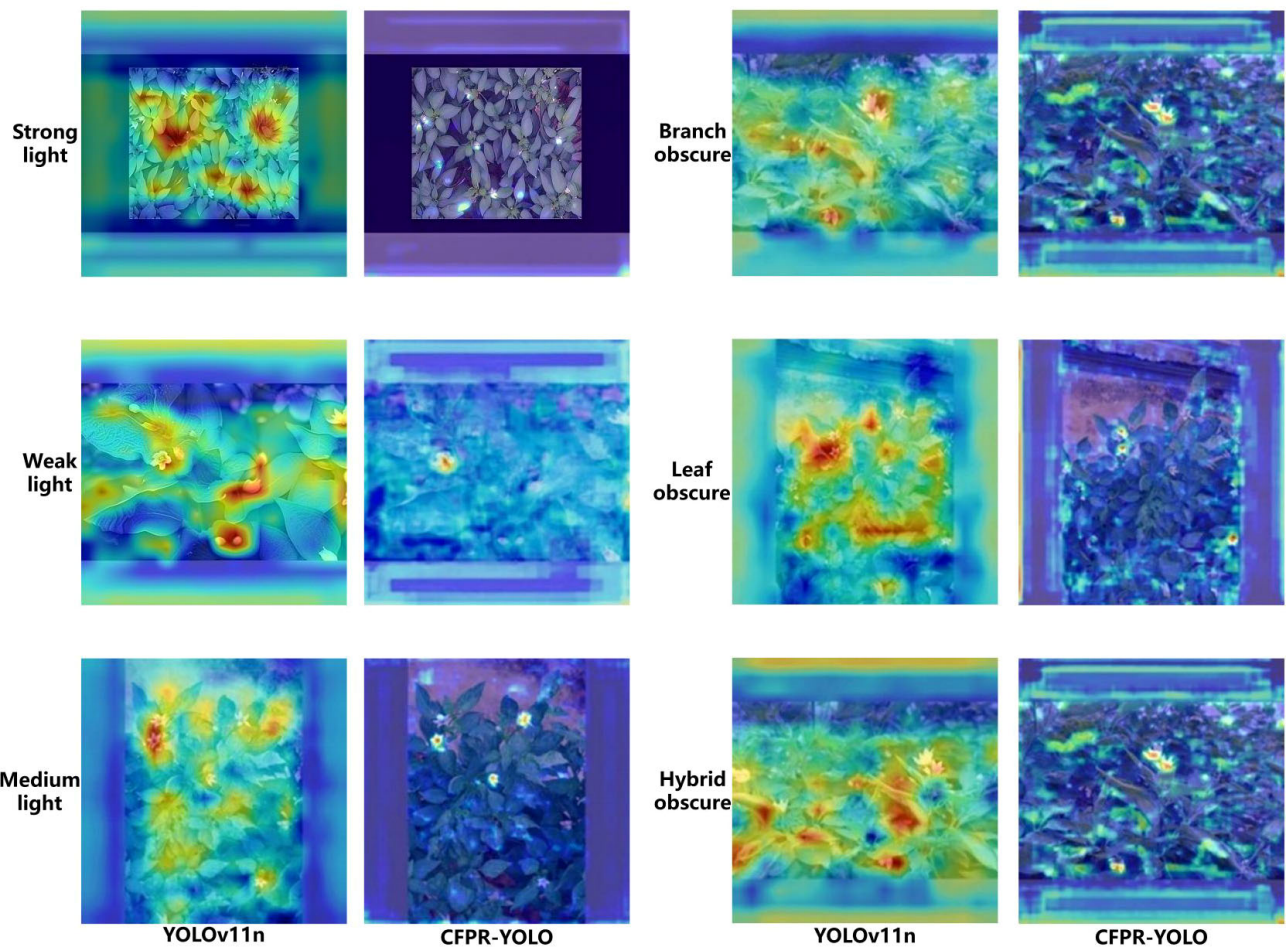
Comparison of visual detection effect of each model under different scenes.

To visually validate the detection robustness of the CFPR-YOLO model in complex environments, this study selected chili flower images from representative natural scenes for comparative analysis. These scenarios included different light intensities (high, medium, and low) as well as varying degrees of occlusion (leaf occlusion, stem occlusion, and mixed occlusion). Using the gradient-based visualization method Grad-CAM (Gradient-weighted Class Activation Mapping) Xiong et al. (2025), heat maps were generated for chili flower pose recognition, as illustrated in **Figure 17**. In these heat maps, red regions indicate areas with higher contributions to target detection, while blue regions represent lower contributions. The results show that, compared with the original YOLOv11n model, CFPR-YOLO produces heat maps that more precisely highlight the core regions of chili flowers, with stronger responses at critical parts such as petal edges and stamens. In contrast, the original YOLOv11n model displays deeper red activations on leaves, stems, and other non-target regions. Furthermore, across diverse lighting and occlusion conditions, CFPR-YOLO consistently and stably emphasizes the main flower regions while effectively suppressing background interference. By comparison, the original model demonstrates scattered heat distributions, weak responses in essential areas, and greater susceptibility to environmental distractions.

This outcome not only verifies the enhanced algorithm’s ability to extract edge features of chili flowers more effectively but also provides comprehensive evidence of the model’s superior detection robustness and environmental adaptability in complex scenarios.

### 3.5 Deployment experiments

To avoid ambiguity among different experimental settings, the deployment-related evaluations in this study were divided into three parts: (1) local GUI visualization experiments based on a PC platform, (2) edge-side inference performance tests on the NVIDIA Jetson AGX Orin platform, and (3) simulated robotic pollination experiments under controlled indoor conditions.

**Figure 18:**
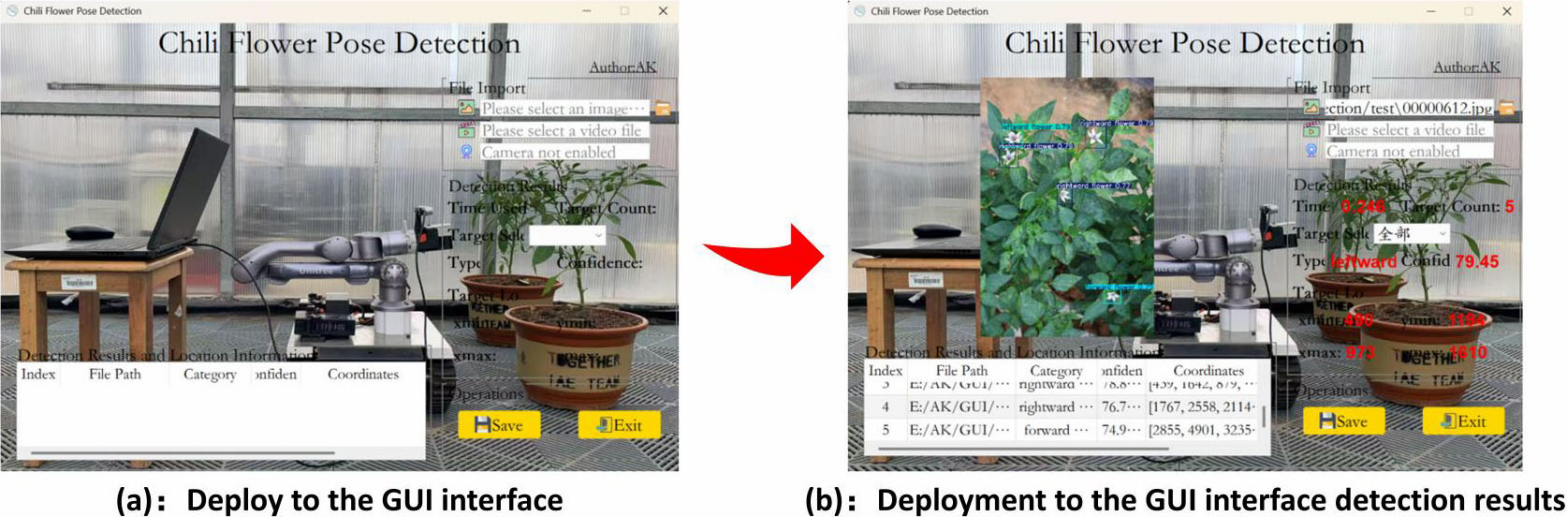
PySide5-based GUI deployment interface.

Considering the common challenges of limited computing power and energy constraints in smart agricultural pollination embedded devices, this study evaluated the feasibility and practical effectiveness of deploying the proposed CFPR-YOLO model on edge devices. To achieve this, a visual GUI based on PySide5 was designed and integrated into the system (As illustrated in **Figure 18**). The interface provides users with an intuitive and interactive platform for model inference and result visualization. Its functionalities include selecting image or video inputs, real-time monitoring of detection outcomes (such as target count, categories, confidence levels, and positional coordinates), and dynamic display of processing time per image.

The system is capable of performing real-time analysis of chili flower images captured by the camera, delivering accurate classification and pose estimation results. This design significantly reduces operational complexity, thereby enhancing the practicality of the model in real-world agricultural applications.

The deployment test results demonstrate that the CFPR-YOLO model delivers favorable overall performance within the GUI environment. The mean processing time per image is 0.246 seconds, while recognition confidence consistently remains above 0.7.

The reported processing time mainly includes image acquisition, model inference, and visualization output, while excluding robotic arm motion control and communication latency.

In addition, the model sustains image processing within 3 seconds, thereby meeting the real-time perception requirements for intelligent chili flower pollination in complex agricultural environments. The interface not only clearly visualizes detection bounding boxes and statistical data but also provides real-time feedback on the positions and categories of multiple flowers within the image, highlighting the model’s stable recognition capacity under challenging lighting and occlusion conditions.

It should be noted that the GUI experiment was mainly designed to verify the visualization and interaction capability of the proposed system on a local workstation platform, rather than to evaluate robotic deployment performance.

**Figure 19:**
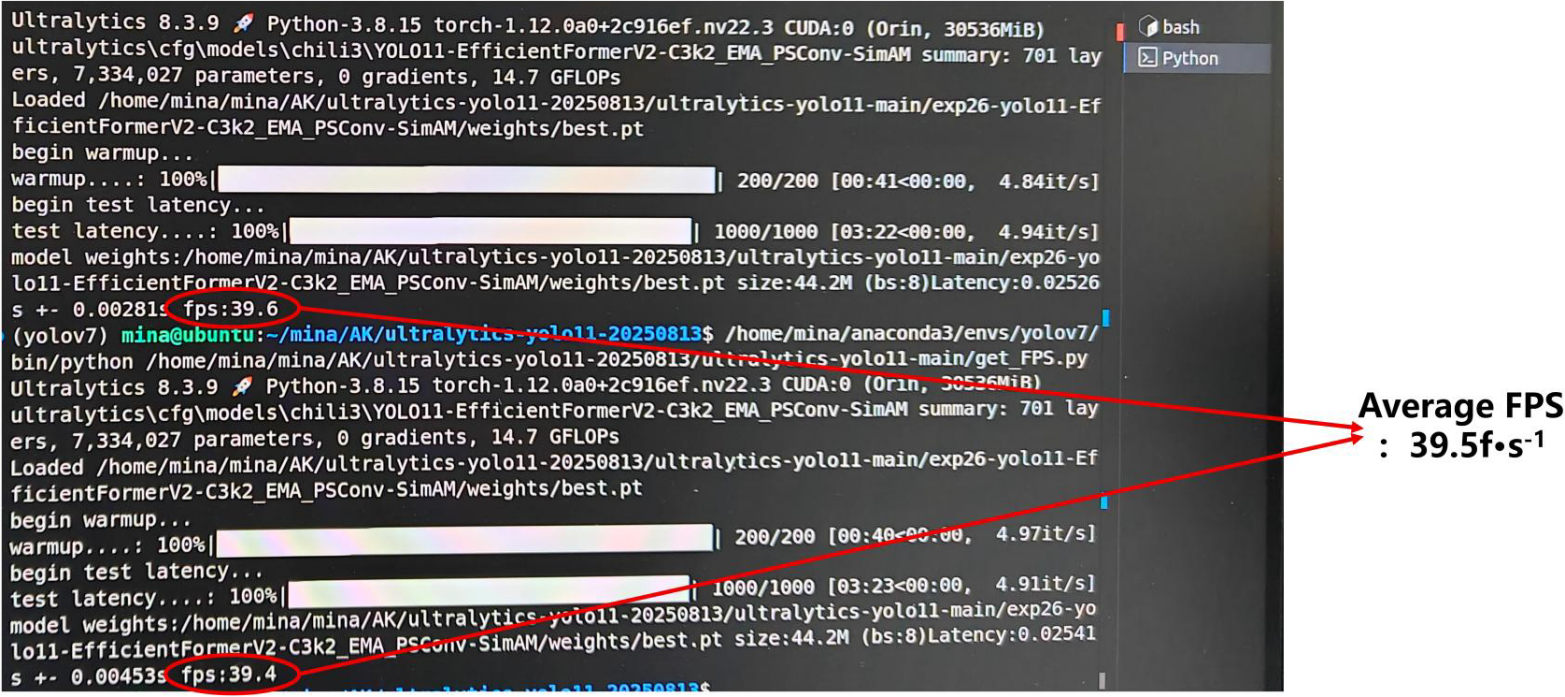
Jetson AGX Orin edge deployment experiment.

While the PC-based GUI validates the model’s efficacy in visual interaction, its practical utility in agricultural scenarios further depends on its runtime efficiency within resource-constrained embedded environments. To evaluate this capability, the trained CFPR-YOLO model was subsequently deployed onto the NVIDIA Jetson AGX Orin edge computing platform (as illustrated in **Figure 19**), thereby simulating the onboard inference environment of an autonomous pollination robot.

The real-time execution logs from the deployment test reveal favorable inference efficiency on the edge device. Operating in FP16 precision mode, the model attained a stable average frame rate of 39.5 FPS.

The FPS value reported here includes image input, neural network inference, and detection result output processes, but excludes manipulator control execution and system communication overhead.

This throughput corresponds to a processing latency of approximately 25 ms per frame, satisfying the real-time visual perception requirements for robotic operation tasks. These results demonstrate that the proposed model maintains stable inference capability on embedded edge hardware and can provide low-latency pose estimation information for robotic perception systems.

### 3.6 Pollination simulation experiments

#### 3.6.1 Visual geometry-based pollination point localization

**Figure 20:**
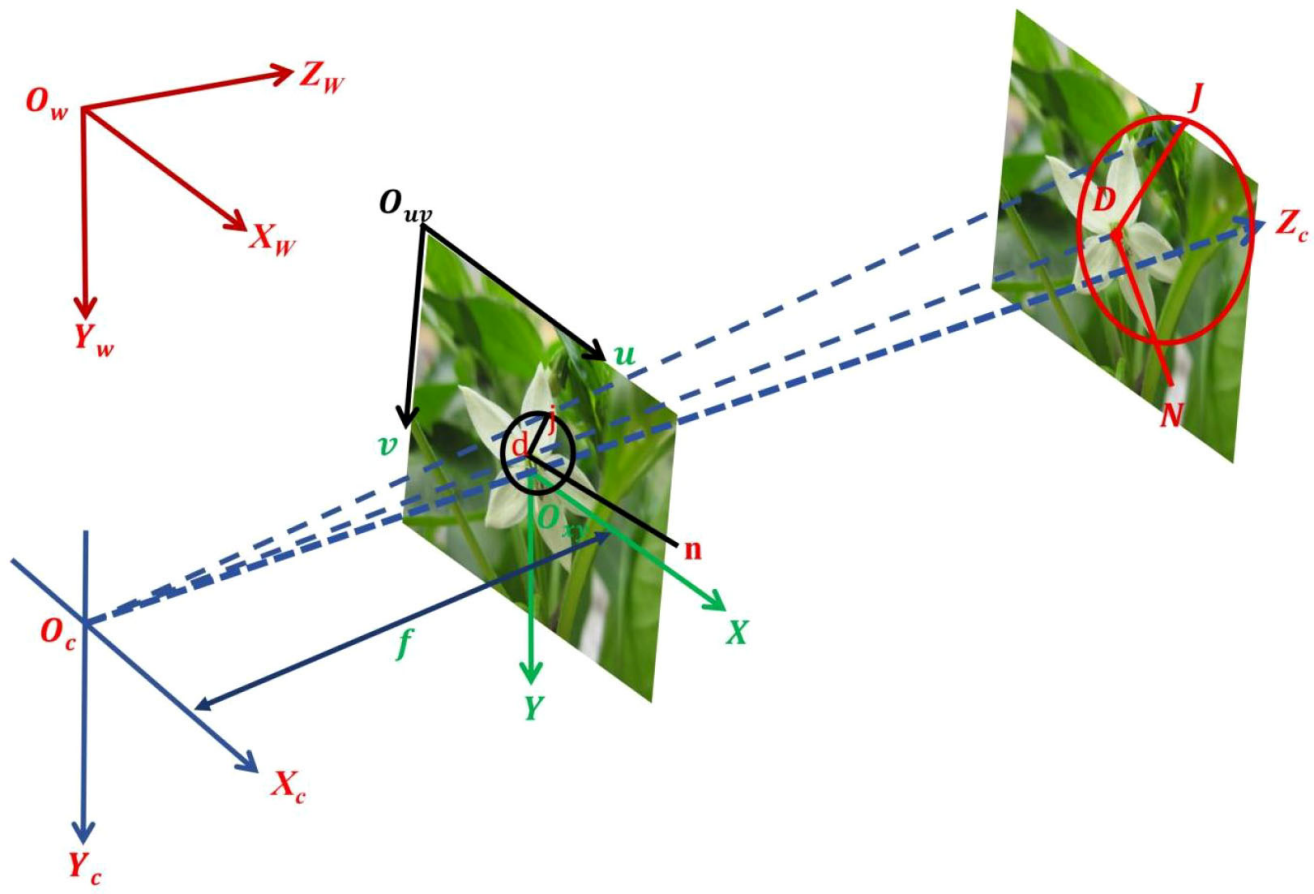
Principle of coordinate transformation for chili flower pollination point localization. *O_w_*represents the world coordinate system (manipulator base frame); *O_c_*is the camera coordinate system; *O_xy_*denotes the image physical coordinate system; and *O_iv_*is the pixel coordinate system. *J* and *N* represent the true pollination center and edge reference point of the chili flower in the world coordinate system, respectively, with an actual physical distance of *D* (mm). Points *j* and *n* are their corresponding projections on the imaging plane, and *d* represents the corresponding distance in the pixel coordinate system.

To guide the robotic manipulator’s end-effector precisely to the pollination target, a mapping model from the 2D image plane to the 3D operational space must be established. As illustrated in **Figure 20**, the pistil center *j* and the petal edge keypoint *n* of the chili flower are first extracted in the pixel coordinate system *O_iv_*via the target detection and pose estimation network. To convert the pixel distance in the image into the actual operational distance in physical space, a transformation from the pixel coordinate system to the world coordinate system *O_w_*is essential.

Let *J* denote the true pollination point and *N* be the edge reference point of the chili flower in the world coordinate system. The physical distance between them is defined as *D* (representing the flower radius or the pollination operation radius). Let *f* be the focal length, representing the distance from the depth camera’s optical center *O_c_*to the imaging plane, and *Z* be the vertical depth distance from *O_c_*to the target flower plane. Based on the pinhole imaging principle and the similarity of triangles, the scaling factor *P* between the pixel scale and the physical scale can be derived as in Equation 16:

(16) $$P=\frac{Z}{f}$$

where *Z* is obtained directly from the depth camera (mm), and *f* is the intrinsic focal length (mm). In the image physical coordinate system *O_xy_*, the projected length $d_{O_{xy}}$ between the pollination point and the reference point is expressed as in Equation 17:

(17) $$d_{O_{xy}}=\frac{D}{P}=\frac{D\cdot f}{Z}$$

Furthermore, assuming the physical size of a single pixel on the image sensor is *µ* (mm/pixel), the corresponding pixel distance (pixels) in the pixel coordinate system *O_iv_*is calculated as in Equation 18:

(18) $$d=\frac{d_{O_{xy}}}{\mu }=\frac{D\cdot f}{Z\cdot \mu }$$

Through this geometric model, the system can dynamically calculate the target pixel coordinates based on the preset pollination radius D and the real-time depth information Z. This allows for the resolution of the 3D pollination vector in the world coordinate system, providing precise end-effector pose inputs for the manipulator’s inverse kinematics solver.

#### 3.6.2 Pollination simulation experiment

Due to seasonal phenological constraints (i.e., the non-flowering period of chili during the experiment), a high-fidelity simulated scenario under controlled conditions was constructed to validate the system’s operational performance. It should be noted that this setup does not represent real greenhouse environments.

The integrated hardware platform of the pollination robot consists of an omnidirectional intelligent mobile chassis, a 6-DOF collaborative manipulator, a contact-based flexible end-effector (customized brush), and an Intel RealSense D435 RGB-D camera. A green-screen-based background apparatus was used to approximate the chili canopy environment under controlled conditions.

For spatial perception and control, an “Eye-in-Hand” configuration was adopted. The camera was calibrated using intrinsic and extrinsic parameter estimation, and a hand–eye calibration procedure was performed to establish the transformation between the camera coordinate system and the manipulator base coordinate system. This enables mapping from image pixels to 3D spatial positions for robotic control.

**Figure 21:**
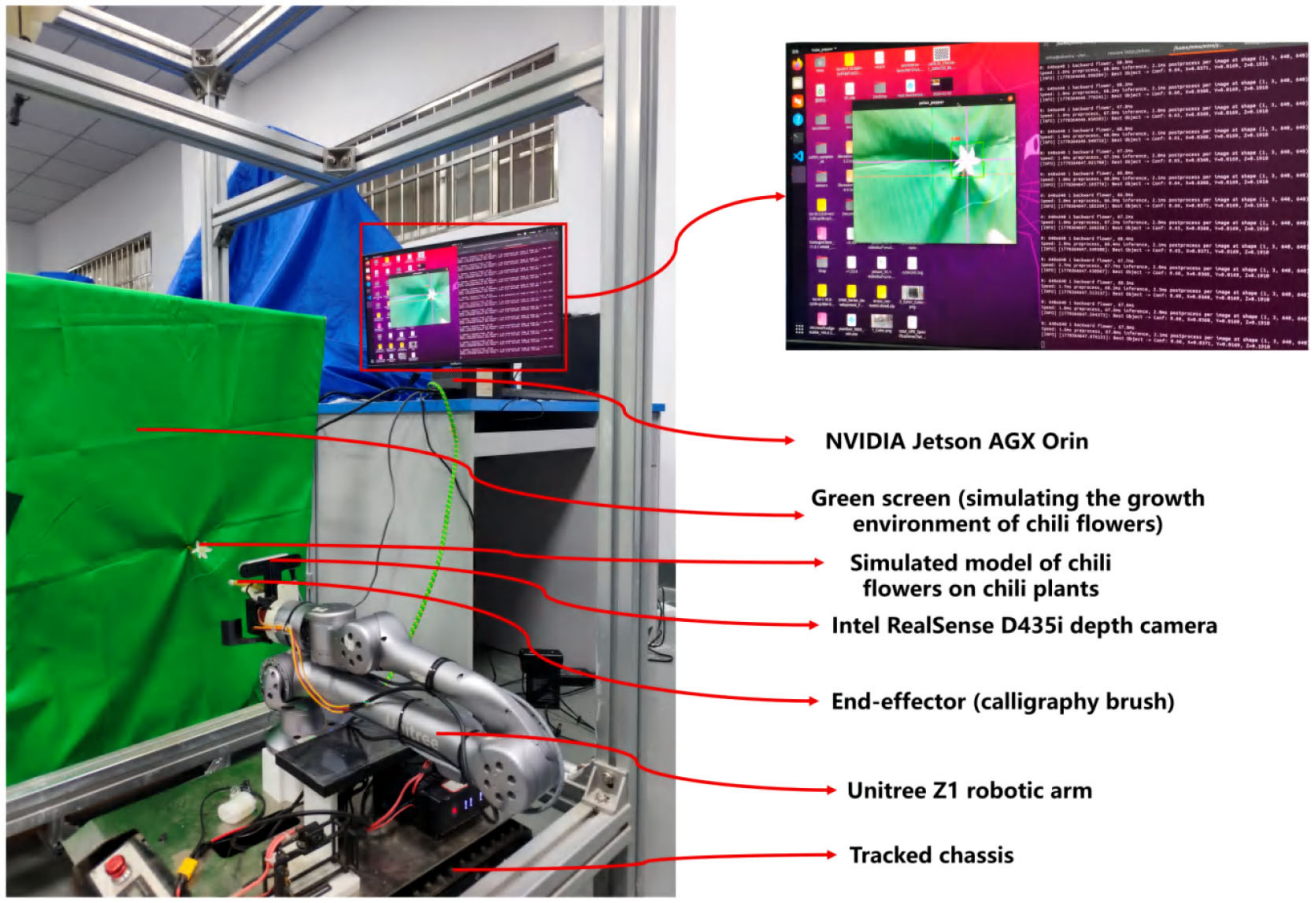
Simulated pollination test scenario.

The overall layout of the simulated pollination experiment is shown in **Figure 21**.

To evaluate deployment performance, the CFPR-YOLO model was deployed on the embedded computing platform of the robot system. Closed-loop automated pollination experiments were conducted on upwardfacing chili flowers under controlled indoor conditions.

**Figure 22:**
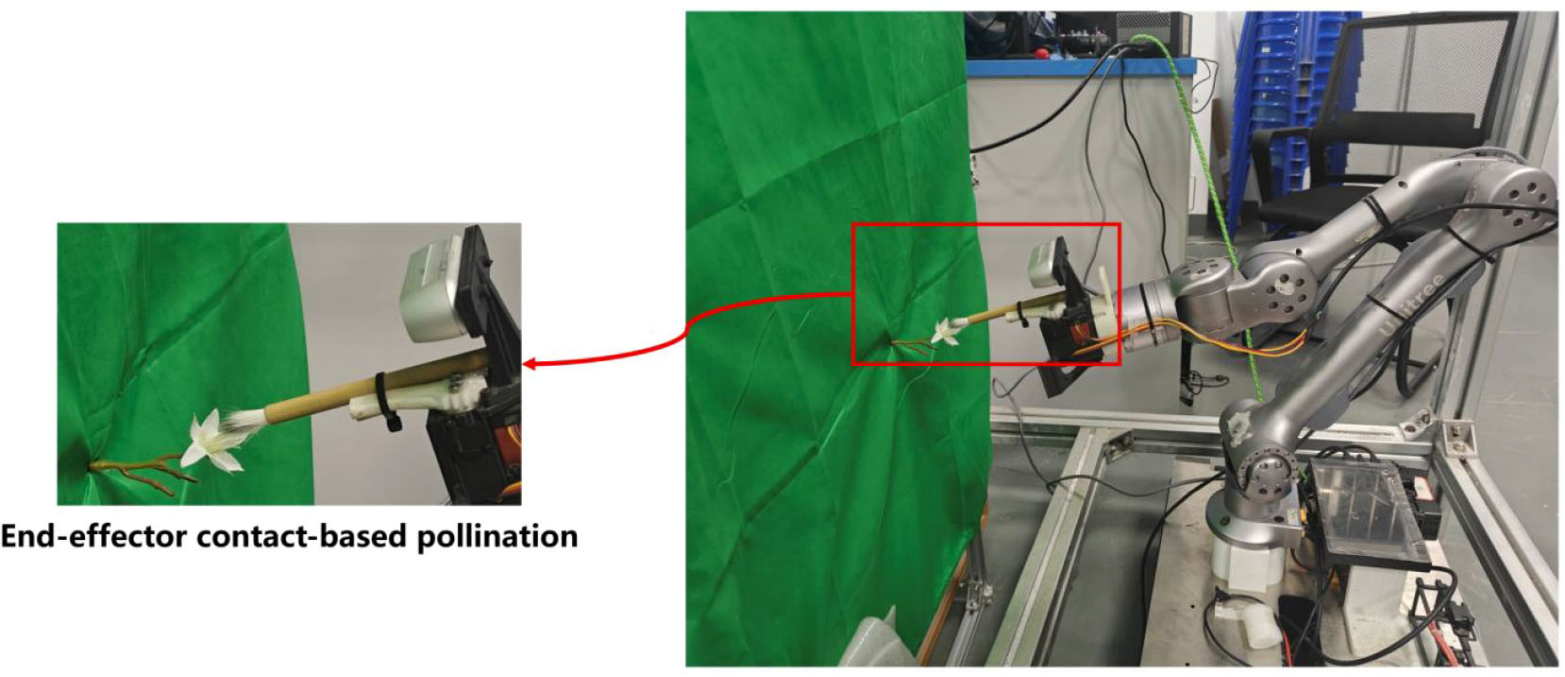
Principle of coordinate transformation for chili flower pollination point localization.

The control process is as follows: the RGB-D camera performs target detection within the field of view; the model predicts the flower category and pose; the detected target is transformed into the manipulator base coordinate system via calibrated transformations; and visual servoing is used to guide the manipulator to approach the pistil region for contact-based pollination. The contact-based pollination effect of the manipulator is shown in **Figure 22**.

To increase variability, the spatial position of the simulated plant was randomly adjusted along the X, Y, and Z axes during experiments to mimic distribution diversity. A total of 100 independent trials were conducted, among which 90 successful contacts with the pistil were achieved, resulting in a success rate of 90.0%.

Failure cases were mainly caused by pose estimation errors under severe occlusion, inaccurate depth measurements under reflective surfaces, and occasional positioning deviations during manipulator motion. These limitations indicate that the current system still faces challenges in complex conditions.

It is important to emphasize that all experiments were conducted under controlled simulated conditions. Therefore, the reported success rate does not yet reflect performance in real greenhouse environments and should be interpreted within the scope of the current experimental setup.

## 4 Conclusion and future work

This study proposed CFPR-YOLO, a lightweight framework for chili flower detection and pose-aware perception under complex agricultural conditions. The method was designed as an edge-deployable visual perception module for intelligent pollination systems and was evaluated on the constructed dataset, deployed hardware platform, and controlled simulation conditions.

By integrating EfficientFormerV2, the C3k2_EMA module, PSConv, and a lightweight attention mechanism, the framework improved the recognition of small, densely distributed, and partially occluded floral targets, while maintaining relatively low computational complexity on the evaluated hardware platform.

Experimental results indicate that CFPR-YOLO achieved strong performance on the self-constructed dataset (mAP@50 of 92.1%) and showed improvements over baseline and mainstream models under the same experimental settings. The model also demonstrated stable performance under varying lighting and occlusion conditions within the collected dataset. Deployment on the evaluated edge platform verified its real-time inference capability under the tested configuration. Furthermore, under controlled simulated pollination conditions, the system achieved a pollination success rate of 90.0% for upward-facing flowers. These results suggest the effectiveness of the proposed framework within the tested dataset, hardware environment, and controlled scenarios.

The contribution of this work lies in developing a perception-to-action pipeline tailored to agricultural engineering informatics, providing reliable visual inputs for robotic pollination, task planning, and precision operations under the tested conditions. However, the findings are limited to the specific dataset, hardware setup, and simulation environment used in this study.

In addition, data augmentation was performed prior to dataset partitioning during the current experimental setup. Although repeated experiments under different random seeds yielded relatively consistent performance trends, augmented samples derived from the same original image may still introduce potential data correlation across subsets. Therefore, the reported results should be interpreted within the scope of the current dataset and experimental protocol. Future studies will adopt augmentation only on the training subset to further improve methodological rigor and evaluation reliability.

Several limitations remain. The framework shows performance degradation in challenging pose conditions (e.g., backward-facing flowers) within the collected dataset. False positives still occur under strong illumination or structurally similar backgrounds. Additionally, the discrete pose representation restricts fine-grained spatial modeling. The system also relies solely on RGB imagery, which limits its capability to capture complex structural information under field conditions.

Future work will focus on improving robustness and applicability under broader conditions. This includes exploring higher-resolution or continuous pose representations, integrating multi-modal sensing data (e.g., depth and spectral information), and further validating the system across more diverse datasets, hardware platforms, and real-world agricultural environments.

## Data Availability

The raw data supporting the conclusions of this article will be made available by the authors, without undue reservation.
